# Blunted anticipation, but not consummation, of food rewards in depression

**DOI:** 10.1016/j.xcrm.2026.102796

**Published:** 2026-05-12

**Authors:** Corinna Schulz, Johannes Klaus, Franziska Peglow, Sabine Ellinger, Anne Kühnel, Martin Walter, Nils B. Kroemer

**Affiliations:** 1 Section of Medical Psychology, Department of Psychiatry and Psychotherapy, University Hospital Bonn, University of Bonn, Bonn, Germany; 2 Department of Psychiatry and Psychotherapy, Tübingen Center for Mental Health, University of Tübingen, Tübingen, Germany; 3 German Center for Mental Health (DZPG), partner site Tübingen, Tübingen, Germany; 4 German Center for Diabetes Research (DZD), Neuherberg, Germany; 5 International Max Planck Research School for the Mechanisms of Mental Function and Dysfunction, University of Tübingen, Tübingen, Germany; 6 Institute of Nutritional and Food Sciences, Human Nutrition, University of Bonn, Bonn, Germany; 7 Department of Psychiatry and Psychotherapy, University Hospital Jena, Jena, Germany; 8 Department of Psychiatry and Psychotherapy, Otto-von-Guericke University Magdeburg, Magdeburg, Germany; 9 German Center for Mental Health (DZPG), partner site Halle-Jena-Magdeburg, Magdeburg, Germany

**Keywords:** major depressive disorder, anhedonia, ghrelin, insulin resistance, taste test, metabolism, gut-brain axis, hedonic capacity

## Abstract

Anhedonia is a core symptom of major depressive disorder (MDD) and includes anticipatory and consummatory reward processes. Yet some measures still equate anhedonia with reduced consummatory pleasure and have rarely been validated against behavioral tasks. In a cross-sectional study, 52 patients with MDD and 51 healthy control participants (HCPs) completed metabolic assessments and repeated food reward ratings, measuring wanting and liking from anticipation to consumption. Patients with MDD showed blunted anticipatory wanting instead of consummatory liking. Once food rewards were available, wanting increased relative to HCPs. More severe anhedonia was linked to reduced anticipatory wanting and steeper increases with consummation, arguing against a consummatory pleasure deficit. Acyl ghrelin was associated with higher reward ratings, while poorer glycemic control related to more severe anhedonia. These findings suggest that anhedonia reflects impaired reward anticipation rather than diminished enjoyment of food rewards and highlight the gut-brain axis as a potential therapeutic target.

## Highlights

• SHAPS-related anhedonia reflects blunted food anticipation but not consummation
• Wanting increases with consummation in anhedonia, arguing against a hedonic deficit
• Poor glycemic control is associated with greater anhedonia
• Acyl ghrelin is linked to elevated food reward ratings

## Graphical abstract

**Figure:**
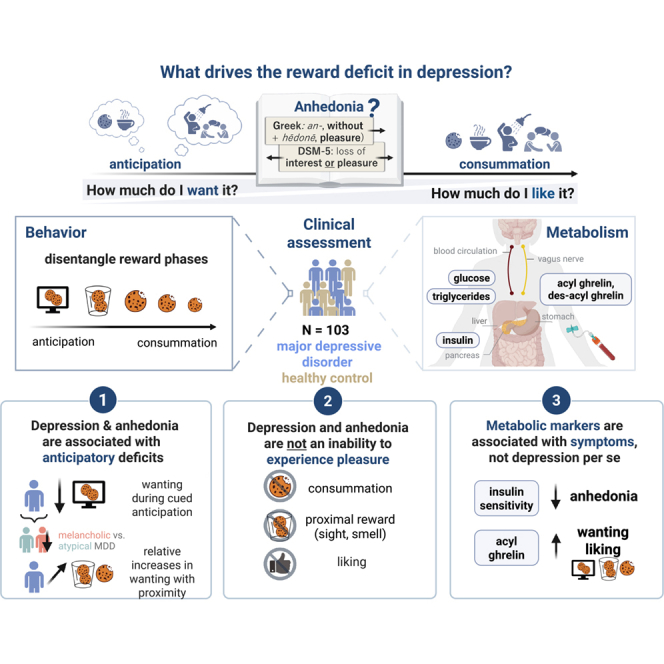

## Teaser

Schulz et al. show that anhedonia in depression, as measured by a scale often equated with loss of pleasure, reflects reduced anticipation of food rewards rather than impaired enjoyment itself. Linking metabolic signals to reward behavior and depressive symptoms highlights the gut-brain axis as a potential target in depression.

## Introduction

As a core symptom of major depressive disorder (MDD), anhedonia is linked to worse treatment outcomes and reduced quality of life, presenting an unmet challenge for therapies.^1^^,^^2^ Recently, the narrow definition of anhedonia as the “decreased subjective experience of pleasure” (as per Ribot’s [1896] definition)^3^ has evolved toward parsing anhedonia into different facets of reward processing, including anticipation and consummation.^3^^,^^4^^,^^5^ In translational research, reward is further dissociated into wanting (i.e., the motivational drive to pursue rewards dominating during anticipation) and liking (i.e., the hedonic pleasure derived from experiencing rewards dominating during consummation).^6^^,^^7^ While ample evidence associates depression with deficits in several facets of reward processing,^8^ comprehensive investigations into anhedonia, which distinguish “when” (anticipation vs. consummation) and “how” (wanting vs. liking) potential deficits manifest, are scarce.^7^^,^^9^ Moreover, patients with MDD often experience opposing changes in appetite echoed in the reward circuit’s functional architecture, suggesting the need to investigate reward deficits in relation to symptoms rather than diagnoses.^10^ In addition to somatic symptoms in MDD, comorbid metabolic disorders^11^^,^^12^ suggest a potential modulatory role of metabolic hormones on reward processing.^13^^,^^14^^,^^15^^,^^16^ However, to harness the potential of metabolic hormones to alleviate reward deficits, a detailed mechanistic understanding of where reward alterations manifest is needed.^17^

Preclinical research has predominantly investigated anhedonia through consummatory reward responses (i.e., hedonic capacity), although the translation of taste-related tests to human research has produced inconsistent results. Seminal research found that rats consumed lower amounts of sucrose and saccharose following chronic stress exposure, mimicking the appetite loss observed in (melancholic) depression.^18^ Since then, preclinical studies have primarily used sucrose intake or sucrose preference tests to assess anhedonia, in which a decreased preference for sucrose is interpreted as reduced liking.^19^^,^^20^^,^^21^ However, conclusive evidence for lower pleasantness ratings of sweet solutions or deficits in gustatory or olfactory function in patients with MDD is lacking.^22^^,^^23^^,^^24^^,^^25^^,^^26^^,^^27^ Instead, emerging evidence suggests that MDD is associated with impairments in multiple stages of reward processing, including anticipation (reduced “wanting”) and consummation (reduced pleasure or “liking”),^7^^,^^8^^,^^28^ with motivation,^29^ decreased coupling of liking and wanting,^30^ or reduced reward learning^31^ observed in anhedonia. Indeed, a recent computational analysis of the sucrose preference test has identified the contribution of several of these (often uncontrolled) reward facets (e.g., wanting) to sucrose preference tests in addition to consummatory liking,^21^ potentially explaining heterogeneous findings. Although several facets, including anticipatory, consummatory, and reward learning deficits^3^^,^^4^^,^^5^ have been implicated in anhedonia, the notion that anhedonia is primarily a consummatory deficit, defined by low hedonic tone, remains pervasive: Many studies continue to describe anhedonia as a diminished capacity to experience pleasure, often using the Snaith-Hamilton Pleasure Scale (SHAPS) as a measure of hedonic tone.^32^^,^^33^^,^^34^^,^^35^ While the SHAPS is a self-report measure that requires individuals to recall or anticipate pleasure across various domains, it is often treated as a proxy for consummatory reward^36^ and is described to lack the assessment of anticipatory pleasure.^37^ This illustrates a key problem: a questionnaire asking the respondent to anticipate and remember reward-related situations is used as if it measures consummatory responses—a generalization that has not yet been validated. Thus, there is a great demand to dissect reward processes behaviorally into facets beyond consummatory liking, ultimately allowing targeted interventions to normalize aberrant reward-related behavior.

Metabolic hormones, such as ghrelin and insulin, play a significant role in reward processing, transcending their role in homeostatic food control.^38^^,^^39^ During fasting, more ghrelin is released, increasing food intake and incentive motivation^40^ via hypothalamic action and possibly vagal projections.^41^^,^^42^ In support of this role, ghrelin has been linked to enhanced food cue reactivity,^43^^,^^44^ food odor conditioning,^45^ alcohol self-administration, and craving^46^^,^^47^ but not food palatability or consummatory reward responses.^48^^,^^49^ However, ghrelin has been mostly investigated with respect to anticipation in humans and rarely contrasting liking and wanting.^17^ Preclinical work further demonstrates that ghrelin amplifies dopamine signaling in the mesocorticolimbic circuit.^38^^,^^50^^,^^51^^,^^52^^,^^53^^,^^54^ Nevertheless, investigations into plasma ghrelin levels in depression have yielded inconsistent results.^55^^,^^56^^,^^57^^,^^58^^,^^59^^,^^60^ In part, such inconsistencies may stem from the heterogeneity of depressive symptoms.^61^ For instance, differences in metabolic dysregulation have been reported between melancholic and atypical depression^62^^,^^63^ and an immune-metabolic subtype of depression for “atypical/energy-related symptoms” has been suggested.^64^^,^^65^ In contrast to ghrelin, insulin increases postprandially, reduces food intake,^66^^,^^67^ and reduces dopamine signaling.^68^^,^^69^^,^^70^ Consequently, intranasal insulin application reduces food preferences, with lower insulin sensitivity attenuating this effect.^71^ Likewise, diminished insulin sensitivity not only weakens the translation of hunger into motivation for rewards^72^ but also serves as an indicator of the efficacy of insulin, as evidenced by its association with the signal-to-noise ratio (i.e., an indicator of the signal effectiveness) of food reward signals in the nucleus accumbens.^73^^,^^74^ Consistent with a metabolic subtype, lower insulin sensitivity has been proposed to contribute to atypical depression.^75^^,^^76^ While MDD frequently occurs with type 2 diabetes,^77^ it has also been linked to low insulin sensitivity in non-diabetic samples from cross-sectional studies,^78^^,^^79^ as well as to metabolic disturbances like elevated triglycerides and increased fasting glucose.^80^ Taken together, metabolic hormones modulate reward processing, with ghrelin potentially enhancing and insulin sensitivity reducing reward responses.

Here, we integrate behavioral, clinical, and metabolic assessments to comprehensively characterize reward processing in depression and anhedonia. Using a behavioral taste test, we repeatedly assessed wanting and liking before and after tasting food snacks, allowing us to move gradually from food reward anticipation to consummation. This design extends previous fMRI research that has shown separable neural activations for anticipation (e.g., ventral striatum^81^) and consummation (e.g., ventromedial prefrontal cortex^82^) of rewards.^83^^,^^84^^,^^85^^,^^86^ Likewise, brain regions recruited during expectation of primary taste rewards as well as anticipatory chemosensation are, in part, dissociable from areas that respond to the receipt of taste rewards and consummatory chemosensation.^87^^,^^88^ However, fMRI designs often confound anticipation, consummation, and learning^73^ and are limited to liquids and highly constrained stimulus sets.^89^^,^^90^^,^^91^^,^^92^ Our behavioral taste test design allows us to move from anticipation to consummation in a more naturalistic setting while including different snacks as food stimuli to improve the generalization. Importantly, we also assessed fasting metabolic hormones (serum/plasma), enabling us to examine how peripheral signals such as ghrelin and insulin sensitivity relate to reward processing and clinical symptoms. We tested three hypotheses: (1) that participants with MDD would show reduced liking or wanting during anticipation and consummation compared to HCPs; (2) that higher anhedonia (SHAPS, as a measure of the hedonic capacity) would be linked to reduced consummatory liking but not wanting; and (3) that heightened reward ratings would be associated with higher levels of ghrelin and lower reward ratings with reduced insulin sensitivity. We found that depression and anhedonia were associated with blunted anticipatory, but not consummatory, responses, while metabolic signals tracked symptoms and behavior, not depression diagnoses themselves. These findings challenge the notion of anhedonia as a generalized hedonic deficit and underscores the potential of investigating the gut-brain axis as a target to treat motivational deficits.

## Results

### Lower wanting but not liking during initial anticipation in MDD

**Figure 1:**
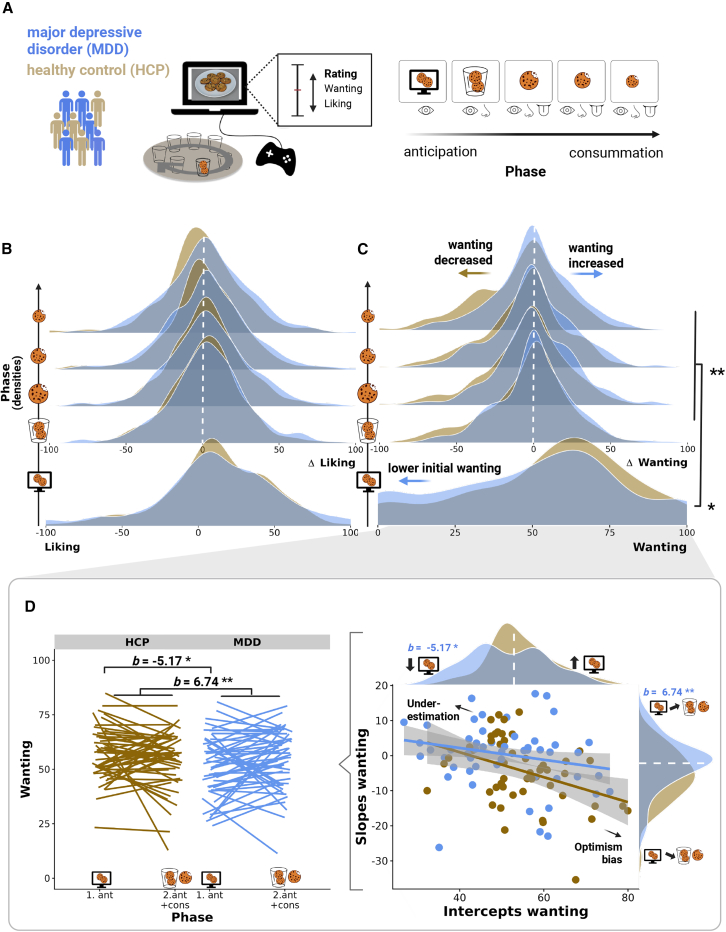
Patients with major depressive disorder (MDD) showed attenuated wanting but not liking during the anticipation of distant food rewards (A) To disentangle reward facets, participants with MDD and healthy control participants (HCP) repeatedly rated liking and wanting of snacks, moving from anticipation to consummation. (B) No group differences in liking ratings (*b* = 0.21; 95% CI: −6.54, 6.96; *p* = 0.95), even after tasting food (*b* = 2.87; 95% CI: −2.10, 7.84; *p* = 0.26). Data are represented as density distributions of individual observations per phase and group. (C) Participants with MDD showed lower wanting ratings during anticipation (*b* = −5.17; 95% CI: −10.10, −0.15; *p* = 0.046). Once the food was proximal, wanting ratings aligned, as patients with MDD reported increases in wanting compared to HCPs (*b* = 6.74; 95% CI: 2.19, 11.28; *p* = 0.004). (D) Individuals with higher initial wanting ratings tended to decrease their ratings with proximal food, reminiscent of an optimism bias in HCPs. Conversely, individuals with lower initial wanting ratings tended to increase their ratings with proximal food, resembling an underestimation bias in MDD. Depicted are individual regression lines (left) and the robust linear regression (with shaded areas indicating 95% confidence intervals) of unbiased (i.e., not including group) random intercepts and slopes derived from mixed-effects models, where differences in wanting ratings are estimated with phase and snack item (right). ∗*p* < 0.05, ∗∗*p* < 0.01. Created with BioRender.com. See also Figure S2.

To disentangle reward facets in depression, we developed a taste test in which participants with and without MDD repeatedly rated food liking and wanting, moving from anticipation to consummation (Figure 1A). Using linear mixed-effect models, we modeled liking and wanting ratings to evaluate group differences throughout the taste test. Since individuals adjusted their ratings once the food was present in front of them (i.e., after an initial anticipatory rating; Figure 1C), a model separating first anticipation (i.e., visual food cues) from consummation (i.e., proximal inspection and sequential tasting) fit the data best (model comparisons; Figure S1). Therefore, we used the two-level phase factor to separate anticipation from consummation (including sight, smell, touch, and taste) for all further analysis. From a theoretical viewpoint, regarding sight, touch, and smell as part of the consummatory phase is reasonable because consummation describes the terminal stage of motivated behavior and does not only refer to “consumption.” Instead, during consummation “motivational stimuli are available at some physical or psychological distance from the organism.”^93^ Importantly, using the three-phase model or the reported two-phase model does not qualitatively change our conclusions (Table S4).

Cookies were the preferred snacks, around 26 points higher on the liking scale and 23 points higher on the wanting scale than on average, and raisins were the least liked snacks, around 17 points lower on the liking and 14 on the wanting scale than on average. A difference of 6.2 points on the liking scale (−100 to 100) corresponds to the difference between “neutral” and “like slightly.” During the first anticipation, participants with MDD reported similar food liking (*b* = 0.21; confidence interval [CI]: −6.54, 6.96; *p* = 0.95; Figure 1B) but lower food wanting (*b* = −5.17; 95% CI: −10.10, −0.15; *p* = 0.046; Figure 1C) compared to HCP (i.e., about five fewer points on the wanting scale [0–100]). During the consummatory phase, participants with MDD reported similar liking (*b* = 2.87; 95% CI: −2.10, 7.84; *p* = 0.26) and no longer indicated lower food wanting (*b* = 1.56; 95% CI: −3.27, 6.40; *p* = 0.53). Consequently, participants with MDD increased wanting with consummation compared to HCPs who decreased wanting (*b*_*Phase*_ = −5.74; 95% CI: −9.04, −2.44; *p* = 0.0009; *b*_*MDDxPhase*_ = 6.73; 95% CI: 2.19, 11.28; *p* = 0.004; Figure 1D). Notably, we did not observe any sex differences or dependencies on medication type. Likewise, inspecting depression subtypes (Figure S2) did not alter the main result of increased wanting with consummation; however, reduced wanting during anticipation was driven by melancholic depression. These results indicate differences in the incentive salience of distant, not proximal, rewards, and there were no differences in pleasure when tasting food in MDD.

### Anhedonia is not associated with reduced consummatory liking

**Figure 2:**
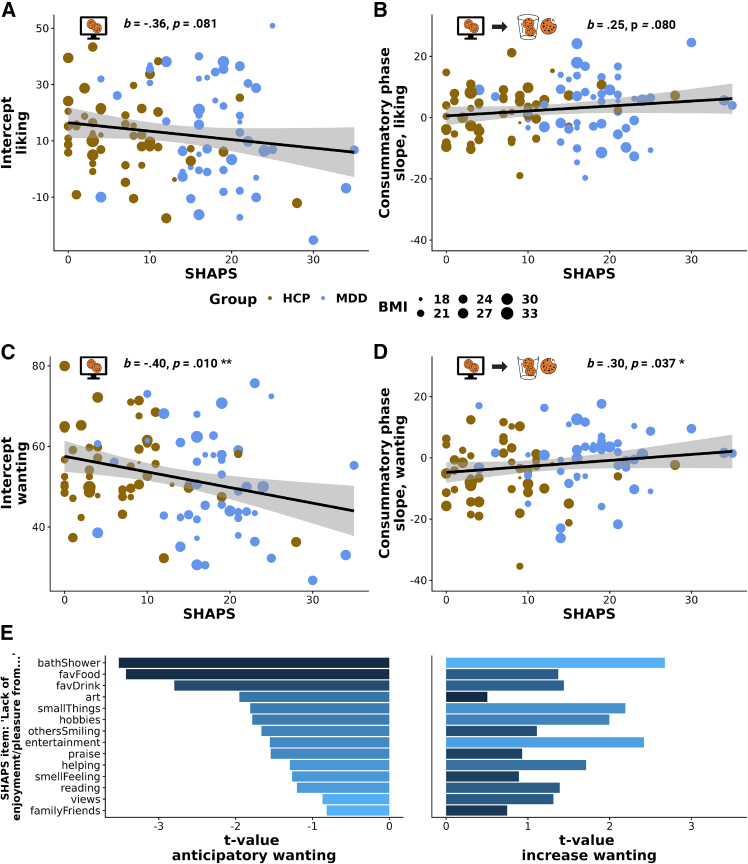
Anhedonia was associated with blunted wanting of food rewards during anticipation but increased wanting with reward exposure (A) Higher SHAPS scores were weakly associated with lower liking during cued anticipation (*b =* −0.36; 95% CI: −0.76, 0.04; *p* = 0.081). (B) Higher SHAPS scores were not associated with increased food liking during the consummatory phase (*b =* 0.25; 95% CI: −0.03, 0.52; *p* = 0.080). (C) Higher SHAPS scores were associated with lower wanting during cued anticipation (*b =* −0.40; 95% CI: −0.70, −0.10; *p* = 0.010). (D) Once food is proximal, higher SHAPS scores were associated with increases in wanting ratings after cued anticipation (*b* = 0.30; 95% CI: 0.02, 0.58; *p* = 0.037). For (A–D), we depict individual intercepts derived from mixed-effects models (excluding SHAPS), with ratings estimated by phase and snack item. Regression lines represent robust linear model fits (rlm), with shaded areas indicating 95% confidence intervals. (E) All SHAPS items were negatively associated with anticipatory wanting (left panel). Similarly, all SHAPS items were associated with increased wanting during the consummatory phase (right panel). ∗*p* < 0.05, ∗∗*p* < 0.01. Created with BioRender.com

After demonstrating reduced wanting ratings during anticipation in depression, we investigated specific associations of ratings during anticipation and consummation with anhedonia (as measured by higher SHAPS scores). In contrast to the alleged reflection of impaired hedonic capacity (i.e., consummatory liking), higher SHAPS scores were associated with reduced wanting (*b* = −0.40; 95% CI: −0.70, −0.10; *p* = 0.010; Figure 2C) but not liking (*b* = −0.36; 95% CI: −0.76, 0.04; *p* = 0.081; Figure 2A) during the anticipation. Since the difference between patients with MDD and HCPs is 11 SHAPS points, this translates into a 4.4-point difference in anticipatory wanting due to differences in anhedonia. Furthermore, participants with higher SHAPS scores showed increases in wanting ratings with consummation (*b*_*SHAPSxPhase*_ = 0.30, 95% CI: 0.02; 0.58, *p* = 0.037; Figure 2D). In contrast, liking ratings did not change (*b*_*SHAPSxPhase*_ = 0.25, 95% CI: −0.03; 0.52, *p* = 0.080; Figure 2B). Single items did not drive associations of anhedonia with reduced anticipatory wanting since we observed negative coefficients for all SHAPS items (Figure 2E). Still, taste-related items (i.e., enjoying favorite food, enjoying a favorite drink) showed the strongest associations with reduced anticipatory wanting. For the interaction with phase, we observed positive associations (i.e., increased wanting with proximal rewards) for all SHAPS items. However, different items showed the strongest association compared to anticipation (Figure 2E). Older individuals (*b* = −0.33; 95% CI: −0.64, −0.01; *p* = 0.047) showed overall lower wanting ratings, but this did not influence the associations with the SHAPS. Depression severity (using the Beck Depression Inventory [BDI]) did not explain lower initial wanting (*b* = −0.15; 95% CI: −0.33, 0.02; *p* = 0.09); however, severity was associated with the observed increases in wanting with proximal food (*b*_*BDIxPhase*_ = 0.20; 95% CI: 0.03, 0.36; *p* = 0.018). As some items from the BDI tap into anhedonia,^94^ we also investigated the BDI anhedonia subscore, partially replicating the pattern for SHAPS (see GitHub). Since lower wanting ratings were associated with SHAPS and melancholic MDD, we inspected the correlation between SHAPS and depression subtype (*r* = −0.082, *p* = 0.56; Figure S3), but these dimensions are largely orthogonal and may contribute independently to altered wanting ratings. Likewise, medication type did not alter the results. Notably, neither depression nor anhedonia was characterized by differences in perceived taste during the consummatory phase (*p*s > 0.11; see GitHub), corroborating that depression and anhedonia are not associated with altered taste perception *per se*. As reported previously by our group, patients with MDD did not differ from HCPs in subjective ratings of metabolic state.^95^

### Moderate-to-strong Bayesian evidence against a consummatory deficit in depression and anhedonia

**Figure 3:**
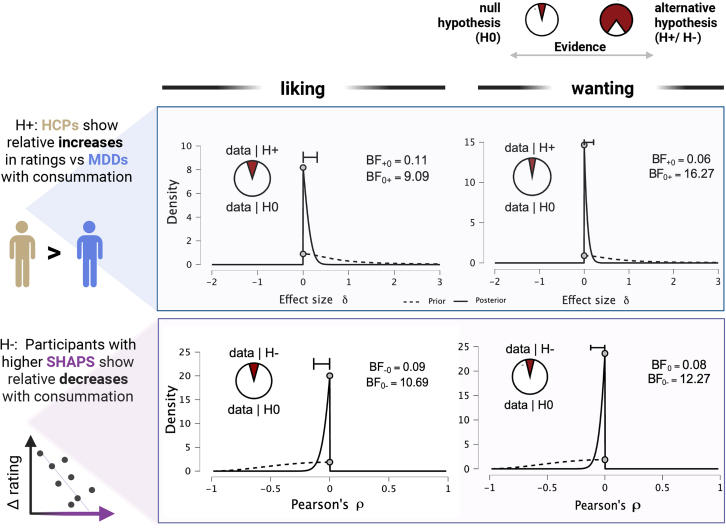
Bayesian hypothesis testing strengthens evidence against the common idea that depression or anhedonia is a consummatory deficit Moderate to strong evidence against the common hypothesis that participants with MDD (vs. HCPs) show relatively reduced liking (moderate) or wanting (strong) during consummation compared to anticipation (top panel; one-sided Bayesian independent samples *t* test). Strong evidence against the hypothesis that higher SHAPS (i.e., lower “hedonic tone”) is associated with stronger liking or wanting decreases during consummation (bottom panel; Bayesian Negative Correlation). BF = Bayes factor (with levels of evidence: 1–3 anecdotal, 3–10 moderate, 10–30 strong). A probability wheel on an area of size 1 represents the BF10, respectively. Created with BioRender.com. See also Figures S4 and S5.

To evaluate the strength of evidence against consummatory reward deficits in patients with MDD and anhedonia provided by our study, we calculated Bayes factors (BFs) for the hypothesized relative decrease in ratings with consummation (Figure 3). The observed increases in wanting provide strong evidence against an alleged consummatory deficit in MDD (BF0+ = 16.27; i.e., 16× more likely to occur if we do not assume decreases with consummation) and in association with anhedonia (BF0+ = 12.27; i.e., 12× more likely if we do not assume decreases with consummation; Figure 3). Likewise, the absence of differences in liking changes provides moderate evidence against an alleged consummatory deficit in MDD (BF0+ = 9.09; i.e., 9× more likely to occur if we do not assume decreases with consummation) and in association with anhedonia (BF0+ = 10.69; i.e., 11× more likely if we do not assume decreases with consummation; Figure 3). As wanting ratings changed in a direction opposite of expectation (i.e., increased rather than decreased with consummation), we additionally tested for an undirected effect, showing moderate (MDD, BF10 = 4.12) and anecdotal (SHAPS, BF10 = 1.26) evidence that ratings increase with consummation relative to anticipation in MDD and with higher SHAPS (Figure S4). Prior selection did not qualitatively change these results (Robustness checks; Figure S5).

To evaluate the strength of evidence of the observed anticipatory deficit in patients with MDD and anhedonia provided by our study, we calculated BF for absolute ratings during anticipation. The observed lower wanting during anticipation provides anecdotal evidence for an anticipatory wanting deficit in MDD, such that HCPs have higher wanting ratings (BF +0 = 2.19), and the association of lower anticipatory wanting with greater SHAPS provides strong evidence for an anticipatory wanting deficit in anhedonia (r = −0.271; BF −0 = 17.75). The lack of higher liking in HCPs compared to MDDs during anticipation provides moderate evidence in favor of the null hypothesis (BF +0 = 4.90), indicating no difference in anticipatory liking. Likewise, the non-significant association between lower liking and SHAPS provides anecdotal evidence for lower anticipatory liking in anhedonia (r = −0.168; BF −0 = 1.76).

### Metabolic hormones are associated with symptoms, not MDD per se

**Figure 4:**
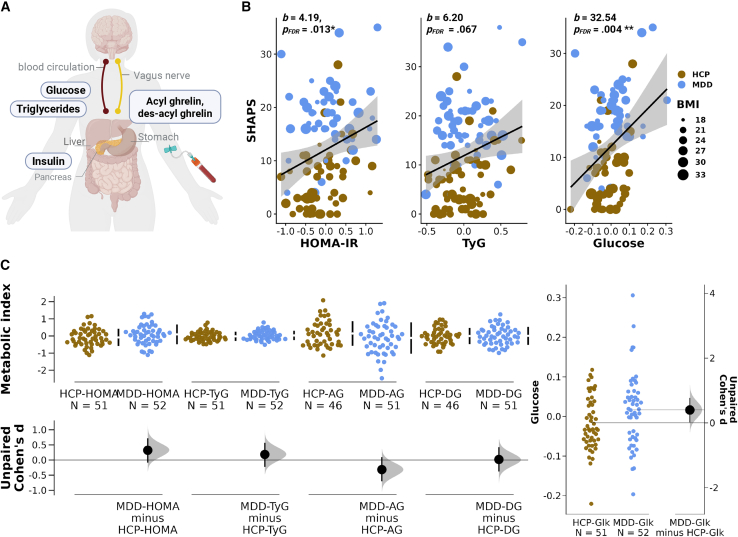
Metabolic disturbances were associated with specific symptom profiles of depression (A) Metabolic parameters were determined after a 12-h overnight fast. Insulin, glucose, and triglycerides were used to determine two indices of insulin resistance: HOMA-IR, and triglyceride index (TyG). (B) Higher insulin resistance (adjusted for BMI, sex, and age) as indexed by HOMA-IR (*b* = 4.19; 95% CI: 1.30, 7.09; *p*_*FDR*_ = 0.013) but not TyG (*b* = 6.20; 95% CI: 0.30, 12.11; *p*_*FDR*_ = 0.067) was associated with SHAPS. Glucose levels were strongly associated with SHAPS (*b* = 33.48; 95% CI: 14.92, 52.06; *p*_*FDR*_ = 0.004). Regression lines represent robust linear model fits (rlm), with shaded areas indicating 95% confidence intervals. (C) Cumming estimation plots show no significant group differences in metabolic indices (HOMA-IR, TyG, Acyl- and Desacyl ghrelin), except glucose levels that were higher in MDD (*b* = 0.03; 95% CI: 0.001, 0.07; *p* = 0.046, but this did not hold when controlling the false discovery rate; *p*_*FDR*_ = 0.22). Raw data points are shown alongside effect size estimates (Cohen’s d; black dots) with bootstrapped 95% confidence intervals. *Note*: all hormonal values were log-transformed and residualized for sex, age, and BMI. ∗*p* < 0.05, ∗∗*p* < 0.01. Created with BioRender.com. See also Figure S6.

Next, we evaluated the association of (corrected) metabolic hormones with symptoms of MDD. As reported in Table 1, (untransformed) metabolic hormone values did not differ between the HCPs and patients with MDD. This finding remained unchanged when using log-transformed values adjusted for age, sex, and BMI and when including age, sex, and BMI as covariates (Figure 4). Fasting acyl ghrelin levels (adjusted for age, sex, and BMI) were not altered in MDD (*b* = −0.27; 95% CI: −0.61, 0.08; *p*_*FDR*_ = 0.22; exp(b) = 0.76; Figure 4C; including depression severity: *b* = −0.55; 95% CI: −1.18, 0.09; *p* = 0.094; exp(b) = 0.58). However, patients with melancholic MDD showed lower ghrelin levels compared to HCPs (*b* = −0.44; 95% CI: −0.85, −0.02; *p* = 0.039; exp(b) = 0.64); however, this did not survive when controlling the false discovery rate (*p*_*FDR*_ = 0.15). Likewise, ghrelin was not associated with SHAPS scores (*b* = −1.63; 95% CI: −3.62, 0.37; *p*_*FDR*_ = 0.14). Fasting levels of des-acyl ghrelin were similar in patients with MDD and HCPs (*b* = 0.007; 95% CI: −0.20, 0.22; *p*_*FDR*_ = 0.94).

**Table 1:** Participant descriptives

Characteristic	Overall *N* = 103	HCP *N* = 51	MDD *N* = 52	Test statistic	*p* value^b^
**Sex, n (%)**	–	–	–	0.78	0.38
Male	42 (41)	23 (45)	19 (37)	–	–
Female	61 (59)	28 (55)	33 (63)	–	–
**Age [years]**	29 0.3 (7.3)	30.5 (7.2)	28.1 (7.4)	1.60	0.11
**Body mass index [kg/m**^**2**^**]**	23.64 (3.25)	23.78 (3.03)	23.50 (3.48)	0.45	0.66
**Acyl ghrelin [pg/m**L**]**^a^	174 (207)	182 (205)	166 (210)	0.38	0.71
**Des-acyl ghrelin [pg/m**L**]**^a^	188 (104)	187 (94)	190 (112)	−0.15	0.88
**Glucose [mg/dL]**	84 (8)	83 (7)	85 (9)	−1.20	0.22
**Insulin [mg/dL]**	62 (38)	55 (28)	69 (45)	−1.90	0.063
**HOMA-IR**	1.89 (1.27)	1.65 (0.94)	2.13 (1.49)	−1.90	0.055
**Triglycerides [mg/dL]**	103 (70)	98 (64)	107 (76)	−0.59	0.56
**TyG**	4.44 (0.29)	4.42 (0.28)	4.46 (0.29)	−0.64	0.52
**BDI**	15.5 (14.4)	3.3 (4.1)	27.6 (9.9)	−16.00	**<0.001**
**SHAPS**	12.3 (8.3)	6.6 (6.0)	18.0 (6.1)	−9.50	**<0.001**
**Anti-depressive medication, n (%)**					
None	79 (77)	51 (100)	28 (54)	–	–
Other	11 (11)	0 (0)	11 (21)	–	–
SSRI	13 (13)	0 (0)	13 25)	–	–

Data are means ± SD, if not indicated otherwise. Abbreviations: HCP, healthy control participants; MDD, major depressive disorder; HOMA-IR, Homeostasis Model Assessment of Insulin Resistance; TyG, Triglyceride-Glucose Index; BDI, Beck Depression Inventory; SHAPS, Snaith-Hamilton Pleasure Scale; SSRI, selective serotonin reuptake inhibitor. See also Table S1.

a Values of acyl and des-acyl ghrelin refer to data of 97 participants. Data of five HCPs and one MDD were missing.

b Pearson’s chi-squared test; Welch two-sample *t* test.

Regarding glycemic control, we observed no group differences in the TyG (*b* = 0.05; 95% CI: −0.06, 0.16; *p*_*FDR*_ = 0.41; exp(b) = 1.05), HOMA-IR (*b* = 0.19; 95% CI: −0.03, 0.40; *p*_*FDR*_ = 0.22; exp(b) = 1.20) or fasting glucose levels (*b* = 0.03; 95% CI: 0.001, 0.07; *p*_*FDR*_ = 0.22; exp(b) = 1.03) in patients with MDD compared to HCPs. This remained unchanged when adding depression severity or depression subtype to the models. Still, patients with MDD showed higher fasting glucose levels (*b* = 0.03; 95% CI: 0.001, 0.07; *p* = 0.046; exp(b) = 1.03). However, this did not survive when controlling the false discovery rate (*p*_*FDR*_ = 0.22). In contrast to MDD, participants with higher SHAPS scores showed higher fasting glucose (*b* = 33.48; 95% CI: 14.92, 52.06; *p*_*FDR*_ = 0.004; i.e., for a 10% increase in glucose, the expected outcome for SHAPS increases by 3.19), lower insulin sensitivity (*b* = 4.19; 95% CI: 1.30, 7.09; *p*_*FDR*_ = 0.013; Figure 4B; i.e., for a 10% increase in HOMA-IR, the expected outcome for SHAPS increases by 0.40), but not higher TyG (*b* = 6.20; 95% CI: 0.30, 12.11; *p*_*FDR*_ = 0.067; i.e., for a 10% increase in TyG, the expected outcome for SHAPS increases by 0.59). Sensitivity analyses showed that the association of anhedonia with HOMA-IR (*b* = 2.53; 95% CI: 0.35, 4.71; *p* = 0.023; i.e., for a 10% increase in HOMA-IR, the expected outcome for SHAPS increases by 0.24) and glucose (*b* = 20.80; 95% CI: 6.66, 34.94; *p* = 0.004; i.e., for a 10% increase in glucose, the expected outcome for SHAPS increases by 1.98) exceeded the effects of MDD since including group in the model did not fully attenuate the associations. The association with the triglyceride index remained insignificant (*b* = 4.21; 95% CI: −0.15, 8.56; *p* = 0.058; Figure 4B), indicative that glucose levels and insulin dominate the association between anhedonia and glycemic control. Again, depression severity or depression subtype did not qualitatively alter these associations. Notably, the association between HOMA-IR and SHAPS was attenuated in melancholic MDD (*b* = −7.11; 95% CI: −12.28, −1.93; *p*_*FDR*_ = 0.040; Figure S6; i.e., for a 10% increase in HOMA-IR in melancholic MDD, the expected outcome for SHAPS is 0.67 lower). We did not observe any sex differences. These results support a link between poor glycemic control and anhedonia in depression.

### Acyl ghrelin is associated with overall higher ratings of food reward

**Figure 5:**
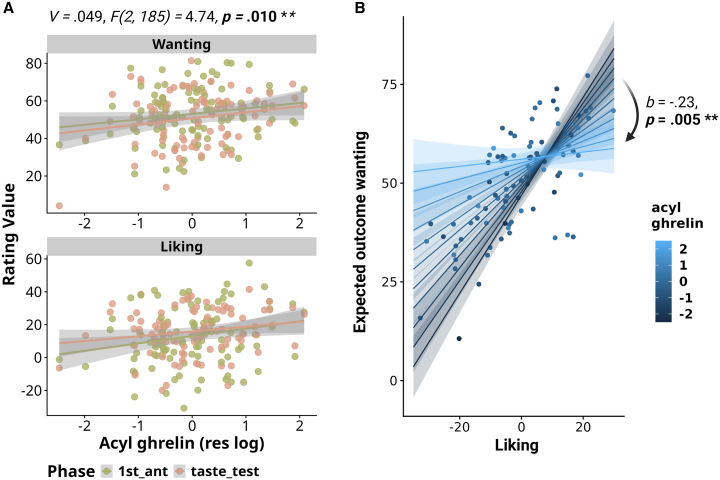
Acyl ghrelin is associated with the coupling between liking and wanting of food rewards (A) Multivariate regression showed that ghrelin was associated with greater food reward ratings and that this association was not specific to liking or wanting [Pillai’s Trace *V* = 0.049, *F*(2, 185) = 4.74, *p* = 0.010]. Regression lines represent robust linear model fits (rlm), with shaded areas indicating 95% confidence intervals. (B) Estimated marginal means of a fitted linear model to estimate average wanting (across phases and snacks), using acyl ghrelin as a fixed effect and its interaction with liking ratings. Ratings of liking and wanting were strongly positively associated, but acyl ghrelin was associated with this coupling. With higher fasting ghrelin levels, food wanting became less dependent on liking (*b* = 0.23; *p* = 0.005; uncorrected). Lines show model-based expected wanting values from the fitted linear model across levels of liking, at different fasting acyl ghrelin levels; shaded areas indicate 95% confidence intervals. ∗∗*p* < 0.01. Created with BioRender.com

Next, we assessed whether metabolic hormones and potential disturbances translate to differential ratings collected during the taste test. To this end, we tested for a multivariate effect of acyl ghrelin and insulin sensitivity on liking and wanting ratings including reward phase, group, and their interaction. Acyl ghrelin was associated with higher ratings overall [Pillai’s Trace *V* = 0.049, *F*(2, 185) = 4.74, *p* = 0.010]. Separate models showed associations for wanting (*t* = 2.88, *p* = 0.005) and liking (*t* = 2.83, *p* = 0.005), supporting a role of ghrelin in incentive motivation (separate linear mixed-effects models considering the hierarchical structure of the data corroborate these conclusions; see GitHub). Adding depression severity to the model did not alter these associations [Pillai’s Trace *V* = 0.05, *F*(2, 185) = 5.34, *p* = 0.006]. Notably, follow-up analyses showed that higher levels of acyl ghrelin were associated with reduced correspondence between wanting and liking ratings (*b*_*likingxGhrelin*_ = −0.23; 95% CI: −0.39, −0.07; *p* = 0.005 (uncorrected); i.e., for a 10-unit increase in liking, the expected outcome for wanting is 0.22 lower for a 10% increase in ghrelin than no change in ghrelin), pointing to a potential shift in the integration of incentive salience and hedonics, such that with higher acyl ghrelin levels less liked food rewards are wanted more (Figure 5B). This interaction did not change when adding group and depression severity to the model. Inspecting the covariates showed that females (*b* = −4.02; *p* = 0.028) and older individuals (*b* = −0.33; *p* = 0.009) showed overall reduced wanting. Follow-up analyses showed this is attenuated in older participants with higher ghrelin (age; *b* = 0.59; 95% CI: 0.21, 0.90; *p* = 0.002 [uncorrected]). In contrast, fasting levels of des-acyl ghrelin showed weaker and non-significant associations with wanting and liking, and we did not observe associations between HOMA-IR with wanting and liking (for full model output, see GitHub).

## Discussion

An improved distinction between failing to seek pleasurable activities and not enjoying them holds actionable implications for treating anhedonia as a cardinal symptom of MDD.^4^^,^^5^ Here, we combined comprehensive clinical, behavioral, and metabolic assessments to localize reward dysfunction in MDD and gauge the potential for interventions targeting the gut-brain axis.^13^^,^^17^^,^^38^^,^^96^ First, we show that patients with MDD and anhedonia primarily experience reduced anticipatory wanting for food rewards. In contrast to the conventional notion that anhedonia is an inability to experience pleasure, we found no differences in anticipatory or consummatory liking and even relative increases in wanting during consummation. Second, our design shows that reward deficits are marked for distal (i.e., cued anticipation) but not proximal (i.e., sight, smell, and taste) rewards, contributing to an improved mechanistic understanding of anhedonia as a motivational deficit. Third, we show that peripheral levels of metabolic hormones are associated with specific aspects of reward function rather than MDD *per se*. Lower insulin sensitivity and higher glucose levels were associated with anhedonia, whereas higher fasting acyl ghrelin levels were associated with higher wanting and liking ratings. Since ghrelin levels were lower in melancholic MDD, it is plausible that altered gut-brain signaling may contribute to motivational symptoms in melancholic MDD experiencing loss of appetite and weight. Our results corroborate the role of reward anticipation in anhedonia and highlight reward proximity and metabolic health as factors for future translational work. Crucially, increases in wanting in patients with MDD and anhedonia during reward consummation provide strong evidence against the hypothesized deficit in hedonic capacity and call for a revision of the term “anhedonia.”

Our findings extend previous work on impaired reward processing in depression by disentangling two crucial phases often investigated separately before^9^: anticipation and consummation. Blunted wanting ratings during anticipation might reflect lower incentive motivation in MDD, reducing the tendency to approach a reward (“wanting”^97^) despite comparable ratings of pleasantness and taste quality. Subjective ratings of wanting have been linked to the recruitment of core regions within the reward circuit^98^^,^^99^ and dopamine neurotransmission for food cues during anticipation,^100^^,^^101^ indicative of incentive motivation to pursue rewards. In depression, blunted recruitment of the reward circuitry during anticipation of incentive cues has been reported,^102^^,^^103^^,^^104^^,^^105^ albeit not consistently,^106^ supporting that the desire for rewards might be altered in MDD. This mechanism may contribute to symptoms of melancholic MDD, which has been associated with the failure to develop a biased response for more frequently rewarded stimuli^107^ and deficits in reward anticipation during a slot machine task.^15^ Accordingly, within the MDD group, we found reduced wanting in the melancholic subtype. Crucially, anhedonia was more strongly associated with blunted wanting (vs. liking) ratings during anticipation, followed by larger increases in wanting during consummation, thereby contradicting the conventional notion that anhedonia reflects an inability to experience pleasure.^4^^,^^108^ Despite the emerging work emphasizing anticipatory and learning-related deficits in MDD in the past decade,^29^^,^^30^^,^^31^ one would still not hypothesize that ratings would increase rather than decrease from anticipation to consummation. Crucially, by using Bayesian statistics, we quantify the strength of evidence provided by our data^109^^,^^110^ and provide experimental moderate to strong evidence against the prevalent idea of a consummatory deficit. This is in line with a recent study showing that anhedonia is associated with reduced striatal reward anticipation but not consummation.^111^ Identifying impaired mechanisms at the individual level is crucial to improve treatments of dysfunctions. For example, anticipatory deficits require different CBT modules than consummatory deficits and may benefit more from behavioral activation therapy^112^ or enhancing episodic future thinking.^113^ Relatedly, tobacco withdrawal can lead to a pharmacologically induced state of (transient) anhedonia,^114^ and a randomized controlled trial found behavioral activation, which encourages planning and engaging in enjoyable activities, improved quit rates and reduced depressive symptoms.^115^ As anhedonia questionnaires inherently assess the recollected experience of distant rewards, they reflect subjective representations of motivational value or negativity bias.^116^ Consequently, they align more closely with processes involved in cued anticipation rather than direct hedonic experiences. In support of this notion, our results argue against using questionnaires, such as the SHAPS, as measures of hedonic capacity.^117^ Instead, our presented results suggest that behavioral assessments provide more nuanced insights into reward deficits that may better guide future translational work than questionnaires alone.

One strength of our study design is that it resolves intra-individual changes across phases, pinpointing lower food anticipatory wanting in patients with MDD and anhedonia. In principle, two processes may explain the group differences in anticipation but not consummation: (1) overestimation of reward value during anticipation in HCP^118^ or (2) underestimation of reward value during anticipation in MDD.^119^ Our findings support both processes: while wanting decreased in HCPs during consummation, it increased in patients with MDD. Crucially, larger corrections of an initial negative bias were associated with anhedonia, substantiating that anhedonia is primarily related to altered motivational reward anticipation.^29^ Our findings also argue against a mechanistic deficit in reward learning that drives anhedonia,^31^ as the differences in wanting ratings faded already in the mere presence of the rewards. Since beliefs about the distance to a reward or desirable state may dictate wanting and instrumental motivation,^120^ participants may differ in their reward-related expectations, irrespective of momentary enjoyment during consummation. For instance, internal beliefs that increase the perceived distance of a reward might reduce hedonic experiences.^121^ Accordingly, negative biases in patients with MDD concerning rewards have been reported before with diverse paradigms.^119^^,^^122^^,^^123^ Taken together, blunted reward anticipation might be explained by perceived reward distance more than by a failure to experience or learn from rewards. Increased reward proximity is targeted by behavioral activation therapy, which augments the exposure to rewards. However, behavioral activation therapy has led to heterogeneous results, possibly because it does not address the negative bias during anticipation effectively and instead capitalizes on reward responsiveness.^112^^,^^124^ Therefore, additional refinements are necessary to treat anhedonia more effectively.

By combining precision-oriented clinical and behavioral assessments with metabolic profiling, our study illustrates the potential of metabolic hormones to modulate reward responses.^15^^,^^16^^,^^17^ In line with the comorbidity between MDD and type 2 diabetes,^77^ lower insulin sensitivity and higher fasting glucose levels were strongly associated with anhedonia. Likewise, hyperglycaemia, diet-induced changes in insulin signaling, and knockout of insulin receptors facilitate depression and anhedonia-like behavior in rodents.^69^^,^^125^^,^^126^^,^^127^ However, we did not observe altered metabolic hormone concentrations in MDD compared to HCPs when adjusting for age, sex, and BMI, supporting that primarily anhedonia is associated with metabolic dysregulation.^128^^,^^129^ Fasting levels of acyl ghrelin were not different in depression, but across groups higher ghrelin was associated with higher wanting and liking ratings across reward phases. This is in accordance with (preclinical) studies showing that ghrelin increases food cue reactivity,^43^^,^^44^ food intake,^130^ and greater motivation to work for food^54^ and is involved in augmenting various drug rewards,^46^^,^^131^^,^^132^ mainly via increased dopamine transmission in the mesocorticolimbic pathway.^132^^,^^133^ The association of ghrelin and subjective ratings of wanting and liking across phases of the taste test supports the role of ghrelin in increasing the “appetizer effect,”^134^ as fasting levels of ghrelin have been associated with increases in subjective appetite during the initial stages of meals.^43^ With higher ghrelin levels, wanting ratings were less coupled with liking, indicating that during an energy deficit, the motivation for food rewards is more independent of the hedonic impact.^135^ Given the conflicting evidence on ghrelin’s action in depression,^76^ we do not find clear evidence for lower ghrelin levels in depression or anhedonia. Yet, a stronger contribution of ghrelin to heightened reward function might be of high clinical relevance for anticipatory deficit in food wanting with anhedonia. In contrast, GLP-1 receptor agonists (suppressing appetite) have been suggested to be discontinued in cases of depression onset,^136^ and there is evidence that GLP-1 agonists might increase the risk of depression and other psychiatric disorders,^137^ although patients with depression are excluded from most trials.^136^^,^^138^ Nevertheless, it is also conceivable that the effective treatment of metabolic dysfunctions would help normalize reward responses,^139^ leading to a net improvement in symptoms, which may explain why some beneficial effects have been reported in patients with type 2 diabetes.^140^ Thus, capitalizing on metabolic signals might provide better treatments for motivational deficits in depression.^141^^,^^142^

Taken together, we disentangled anticipatory and consummatory phases of reward processing to show that depression and anhedonia are characterized by blunted reward anticipation rather than an inability to derive pleasure from rewards. Crucially, we found that wanting already improves with the proximity of the food reward, pointing to a motivational deficit that is corrected by larger consummatory increases compared to healthy individuals. In line with the motivational role of ghrelin, our results highlight that altered gut-brain signaling may contribute to blunted reward function across phases, which may contribute to the symptoms of melancholic MDD. To conclude, precision-oriented behavioral assessments may pave the way toward optimized treatments of reward deficits to improve the quality of life of patients with anhedonia. Based on our findings, encouraging patients with MDD to deliberately experience rewards by removing potential motivational roadblocks may provide a surprisingly straightforward improvement that can be incorporated into cognitive-behavioural treatment modules.

### Limitations of the study

Despite notable strengths, several study limitations should be addressed in future work. First, parsing reward behavior into different facets revealed differences between cued anticipation and consummation, but the strong effect of reward proximity on group differences was unexpected. Future research may systematically vary additional components such as proximity and probability or certainty of the reward outcome.^29^^,^^143^^,^^144^ Second, we assessed inter-individual differences in fasting levels of hormones and meal-related changes in hormone levels after the consummatory phase could reveal additional contributions to the regulation of reward function. Relatedly, the absence of differences in peripheral hormone levels does not preclude differences in central levels or central sensitivity.^75^^,^^145^ Interventional studies administering insulin or ghrelin will help substantiate the link between metabolism and reward processing in MDD. Third, the taste test consisted of palatable food snacks that might not generalize to other rewards. While we did not find specific associations of reward ratings with single items of the SHAPS, future studies will need to test whether an anticipatory deficit instead of a consummatory deficit pertains to other rewards (e.g., social rewards), as has been shown in schizophrenia,^146^ potentially using ecological momentary assessments. Fourth, we assumed linear relationships in our sample since it was metabolically healthy. However, non-linear relationships are common in medicine, and plausible and larger studies should test for non-linear associations, especially if they include patients with metabolic disorders (e.g., type 2 diabetes). Fifth, given the heterogeneity of MDD symptom profiles,^61^ our use of an atypical balance score captured well-documented differences among patients. Still, the atypical balance score does not consider the DSM-5 mood reactivity criteria for atypical depression, and heterogeneity of the construct is hindering evidence synthesis.^147^ Thus, future research could use symptom networks instead,^148^ which require much larger samples though. Although our repeated-measures design allows for fine-grained assessment of reward phases, the cross-sectional nature of the study limits causal inference regarding the temporal relationship between reward deficits and depressive symptoms. Future longitudinal and interventional studies are needed to establish directionality. Lastly, study participation introduces potential selection bias, as individuals who choose to participate may differ systematically from those who do not, which might affect the generalizability of the findings. Information bias may arise from the self-reported outcomes as participants’ ratings of food liking and wanting could be influenced by various reporting biases. Despite our efforts to control for confounders (i.e., BMI, age, and sex), residual confounding may arise from unmeasured or inadequately measured variables, potentially influencing the observed associations.^149^ Relatedly, future (larger) studies may investigate sex-specific effects.

## Resource availability

### Lead contact

Requests for further information and resources should be directed to and will be fulfilled by the lead contact, Nils B. Kroemer (nkroemer@uni-bonn.de).

### Materials availability

This study did not generate new unique reagents or materials.

### Data and code availability

De-identified data have been deposited at GitHub/Zenodo (Zenodo: https://doi.org/10.5281/zenodo.19499946). They are publicly available as of the date of publication. All original code has been deposited at GitHub/Zenodo and is publicly available (Zenodo: https://doi.org/10.5281/zenodo.19499946). Any additional information required to reanalyze the data reported in this paper is available from the lead contact upon request.

## Acknowledgments

We thank Ebru Sarmisak, Anne Schiller, Antonia Schlaich, and Rauda Fahed for help with data acquisition. We also thank Stephanie Ebbinghaus who kindly helped with running the ELISA tests. The study was supported by 10.13039/501100001659DFG KR 4555/7-1, KR 4555/9-1, KR 4555/10-1, and WA 2673/15-1. Figures were created with BioRender.com.

## Author contributions

C.S., formal analysis, visualization, project administration, investigation, writing – original draft preparation, and reviewing and editing. J.K., project administration and investigation. F.P., investigation. A.K., writing – reviewing and editing. S.E., investigation, writing – reviewing and editing. M.W., conceptualization, methodology, and funding acquisition. N.B.K., conceptualization, methodology, funding acquisition, supervision, project administration, validation, and writing – reviewing and editing. All authors read and approved the final version of the manuscript. C.S. and N.B.K. have accessed and verified the data.

## Declaration of interests

J.K. works as a study therapist in a multicenter phase IIb study by Beckley Psychtech Ltd on 5-MeO-DMT in patients with MDD, unrelated to this investigation. J.K. did not receive any financial compensation from the company. M.W. is a member of the following advisory boards and gave presentations to the following companies: Bayer AG, Germany; Boehringer Ingelheim, Germany; Novartis, Perception Neuroscience, HMNC, and Biologische Heilmittel Heel GmbH, Germany. M.W. has further conducted studies with institutional research support from HEEL and Janssen Pharmaceutical Research for a clinical trial (IIT) on ketamine in patients with MDD, unrelated to this investigation.

## STAR★Methods

### Key resources table

**Table:** 

REAGENT or RESOURCE	SOURCE	IDENTIFIER
**Biological samples**		

Human blood samples (fasting)	This study	NCT05318924

**Critical commercial assays**		

Human Acyl Ghrelin ELISA Kit	Bertin Bioreagent (Bertin Technologies); distributed by BioCat	Cat# A05306
Human Unacylated Ghrelin ELISA Kit	Bertin Bioreagent (Bertin Technologies); distributed by BioCat	Cat# A05319
Atellica CH Triglycerides_2 assay	Siemens Healthineers	Atellica Solution CI Analyzer
Atellica IM Insulin assay	Siemens Healthineers	Atellica Solution IM Analyzer
Atellica CH Glucose Hexokinase_3 assay	Siemens Healthineers	Atellica Solution CI Analyzer

**Deposited data**		

De-identified dataset and analysis code	GitHub (neuromadlab)/ Zenodo	Zenodo: https://doi.org/10.5281/zenodo.19499946

**Software and algorithms**		

R statistical software v3.1.3	R Foundation for Statistical Computing	https://www.r-project.org
lmerTest R package (v3.1.3)	CRAN	https://github.com/runehaubo/lmerTestR
JASP (v0.18.3)	JASP Team	https://jasp-stats.org
effectsize R package	CRAN	https://easystats.github.io/effectsize/

### Experimental model and study participant details

This cross-sectional study characterized patients with MDD and HCPs with respect to their subjective ratings of food reward during different reward phases (i.e., repeated ratings gradually moving from anticipation to consummation, analogous to a short-term panel design, enabling us to examine within-participant changes.^150^ A sample size of 50 healthy control participants and 50 MDD patients was calculated beforehand to achieve a high sensitivity (at a power 1-β = 0.80) for moderately sized group differences (d = 0.57, generic r = 0.27).

The sample consisted of 103 participants, matched for age and body mass index (BMI; *M*_*Age*_ = 29.3 ± 7.3 years, *M*_*BMI*_ = 23.6 ± 3.3 kg/m^2^ [means ± SD]; Table 1), including 52 participants with MDD and 51 HCPs, who had never experienced a depressive episode. Sex was recorded using self-reports. Among the 103 participants, 42 were male, and 61 were female. Sex was included as a covariate in all analyses; however, the study was not powered to investigate sex-specific effects, which should be addressed in future research. Most participants had obtained a high school diploma (“Abitur”; 46%) and were studying and 33% had already obtained a university degree. All individuals interested in participating were screened for eligibility by telephone. Individuals were included if they (1) were between 20 and 50 years old, (2) had a body mass index (BMI) between 18.5 kg/m^2^ and 30.0 kg/m^2^. They were excluded if they (1) ever met criteria for schizophrenia, bipolar disorder, severe substance dependence or neurological condition, or for HCP, mood or anxiety disorders, (2) met criteria for eating disorders, obsessive-compulsive disorder, trauma, and stressor-related disorder, or somatic symptom disorder within the last 12-month, (3) took medication (except anti-depressive medication for MDD), or suffered from illnesses that influenced body weight, (4) for female individuals if they were pregnant or nursing at the time. For the MDD group, individuals needed to fulfill DSM-5 criteria for MDD at screening. Individuals with comorbid anxiety disorders were also included due to the high comorbidity^151^ (participant comorbidities Table S1). To improve generalizability, we imposed no restrictions on treatment type (e.g., psychotherapy, pharmacological, or apps) during the recruitment. However, to minimise confounding effects due to pharmacological changes, we required patients to be on stable medication for at least two months before study participation. Individuals were recruited using flyers and advertisements on social media (Facebook, Instagram) within the area surrounding Tübingen. From 05.10.2021 until 31.05.2023 (time of data freeze), 697 participants were screened on the telephone for initial eligibility for several studies running in the lab, including the one described here. 292 participants were found to be potentially suitable for this study, of which 133 were invited to participate in the study described here. Of these, 20 participants dropped out before their first session, and 3 dropped out during the study (of which 1 dropped out due to exhaustion). Additionally, 7 participants were excluded during the study (5 because they were identified with past or current severe substance abuse, 1 because of concurrent PTSD, 1 because of breastfeeding). The recruitment process resulted in 103 participants out of the 133 invited, yielding a response recruitment rate of approximately 77%. No information on gender, race, or ancestry was collected in accordance with local regulations and data protection policies. All participants were German-speaking.

Before inclusion, all individuals signed written informed consent. All procedures were approved by the local Ethics Committee of the University of Tübingen, Faculty of Medicine (662/2018BO1), in accordance with the Declaration of Helsinki (as revised in 2008). The compensation consisted of money and food rewards that could be acquired through the tasks (i.e., for full completion of the study, either €50 or 5 credit points + performance-based rewards). The study took place at the Department of Psychiatry and Psychotherapy in Tübingen, Germany.

### Method details

#### Experimental procedure

Participants were invited to the laboratory for two parts: a clinical interview and a behavioral intake session. Due to the different durations of the clinical interview, HCPs usually completed both parts on one day, while participants with MDD completed them on separate days. During the first part, all participants completed the Structured Clinical Interview for DSM-V (SCID-5-CV; First^152^; ∼1.5–2 h for MDD, ∼30 min for HCP). In addition, participants with MDD completed the Structured Interview Guide for the Hamilton Rating Depression Scale with Atypical Depression Supplement (SIGH-ADS; Williams & Terman, 2003). The second part included fasting blood draws and a battery of reward-related tasks on the laptop (∼3.5 h). After a 12 h overnight fast – during which participants were instructed only to consume unsweetened beverages (e.g., water or coffee), participants answered state-related questions on a visual analogue scale (VAS) repeatedly to indicate their current subjective metabolic (i.e., feelings of hunger, fullness, and satiety) and affective state. Blood samples for the determination of acyl and des-acyl ghrelin (EDTA plasma), glucose (fluoride EDTA plasma), insulin (serum), and triglycerides (lithium heparinised plasma) were taken upon arrival by using Monovettes (Sarstedt, Nümbrecht, Germany). Afterward, information was recorded on the participant’s last meal and drink, on anthropometric data (e.g., body weight and height), and, in the case of female participants, on the menstrual cycle phase.^95^ Then, participants started with a battery of reward-related tasks. As part of this battery, they completed a food cue rating task (∼15 min;^153^)) and, ∼50 min later, a taste test (∼25 min). During the study session, individuals were provided with water *ad libitum*. The session concluded with participants receiving their financial compensation.

#### Measures

##### Hormone levels

Monovettes were transferred to the Central Laboratory of the Institute of Clinical Chemistry and Pathobiochemistry of the University Hospital Tübingen for analysis of glucose, insulin, and triglycerides in plasma or serum. Glucose was determined in sodium fluoride plasma using an enzymatic test kit (Atellica CH Glucose Hexokinase_3; Atellica Solution, CI analyser), insulin in serum using an immunological assay (Atellica IM Insulin; Atellica Solution, IM Analyzer) and triglycerides in lithium heparin plasma using of an enzymatic assay (Atellica CH Triglycerides_2, Atellica Solution, CI Analyzer; Siemens Healthineers, Eschborn, Germany; within-laboratory precision for glucose ≤2.2%, for insulin ≤10%, and for triglycerides ≤4.0% according to manufacturer). Plasma samples for analysis of ghrelin were obtained from K3E-EDTA Monovettes immediately by centrifugation of the blood samples at 4°C with 2000 g for 10 min. Then, 500 μL of plasma was transferred into two cooled cryo tubes (Thermo Scientific Nunc) each and 50 μL of cooled 1 M hydrochloric acid (HCl) in plasma to acid ratio of 10:1 was added to each tube to prevent ghrelin from deacetylating. The tubes were immediately capped, gently reversed, and cooled at −20°C before they were stored at −80°C (after 24 to 48 h). After completing the trial, the frozen samples were transferred on dry ice to the University of Bonn. The concentration of both acylated and unacetylated ghrelin was determined by using ELISA kits (#A05306 and #A05319; both from Bertin Bioreagent, Bertin Technologies, Montigny-le-Bretonneux, France; distributed by BioCat, Germany) at the Institute of Nutritional and Food Sciences, Human Nutrition.

##### Food cue reactivity and taste test

To assess different facets of reward processing, we used a food cue reactivity (FCR) task (distal sight;^153^) and a taste test paradigm (proximal sight/smell and tasting; Figure 1A). Participants rated how much they liked and wanted 7 snacks during 5 phases (1^st^ food cues, 2^nd^ proximal inspection of snack and smelling the actual snack, 3^rd^ – 5^th^ repeatedly tasting the snacks). The FCR task is a widely used task to assess food anticipation.^141^^,^^154^ Here, it included the 7 snacks of the taste test among a set of 60 food and 20 non-food images^155^ optimised for visual characteristics (homogenous plate with gray background). Participants were presented with each item for 2 s twice before they rated them using a joystick on an Xbox controller and confirming by pressing the A button.^156^ The rating scale was presented for a maximum of 2.8 s. In separate trials, they rated how much they liked and wanted the items on psychophysically validated labeled magnitude scales.^157^ For liking, participants were asked to compare to all experienced sensations on a vertically labeled hedonic (visual analog) scale. Liking ratings ranged from −100 (strongest disliking imaginable) to +100 (strongest liking imaginable.^157^; Wanting ratings were acquired using a horizontal scale and ranged from 0 (not wanted at all) to 100 (strongly wanted). The order of stimulus presentation and rating was pseudo-randomized. Repeated food liking and wanting ratings from a previous study in our lab (unpublished; see GitHub) support the validity of these measures: within-measure reliability (e.g., wanting–wanting) was consistently higher than the between-measure correlation (wanting–liking), supporting the empirical separability of the constructs. In addition, the same dataset demonstrated good short-term test–retest reliability for both liking and wanting ratings.

The taste test included 7 snacks that were repeatedly rated during phase 2–5. The snacks were placed into separate glasses arranged in a circle on a wooden turntable. The glasses were prepared with enough material to have a few items each round. However, participants could decide for themselves how much they needed to consume to rate the reward, which was typically one item. Water for rinsing between trials was provided. In addition, the corresponding pictures of the FCR set were shown on a laptop screen. Analogous to the FCR, participants then rated the items regarding food liking and wanting. They also rated the snack’s intensity, sweetness, saltiness, and savoriness.^158^ As snacks were used pretzels (399 kcal/100g), NicNac’s (555 kcal/100 g; 527kcal/100 g for the vegan alternative), and bread rings (460 kcal/100 g) as salty snacks, rice crackers (380 kcal/100 g) as neutral snack, and raisins (318 kcal/100 g), chocolate chip cookies (502 kcal/100 g; 491 kcal/100 g for vegan alternative), and strawberry gummies (354 kcal/100 g) as sweet snacks.

This design allows for adjustments to reward ratings with repeated exposure, enabling us to examine within-participant changes^150^ in reward responses over time (from anticipation to consummation) in a controlled setting rather than relying solely on imagined responses as in conventionally used self-report measures. There is substantial evidence from rodent studies and human computational work that liking and wanting are distinct concepts that might act on different timescales and should be studied in concert instead of assuming a unidimensional reward representation.^6^^,^^159^ While liking might serve as an initial and editable estimate about the true, long-run worth of goods, wanting might reflect the true, long-run worth of goods (e.g., nutritive value).^159^ Crucially, both constructs are imaginable to have anticipated and remembered forms.^159^ Therefore, we measure both liking and wanting, during anticipation and consummation, and analyze them as distinct yet interdependent constructs. In addition, we did a sensitivity analysis of a thought experiment where we assume that wanting is dominant during anticipation and liking during consummation and compare how wanting ratings change from anticipation into liking ratings during consummation. Since this requires a common scale to compare both measures, we can *Z* score wanting during anticipation and liking during consummation and calculate their difference. To test whether individuals differ in their change from liking to wanting, we used a linear-mixed model with the difference *Z* score as outcome measure and with Group, Snack, Age, Sex, BMI as fixed effects. This shows that patients with MDD significantly increase their ratings from wanted anticipation to liked consummation (*b* = 0.28 [0.11; 0.46], *p* = 0.0019; *r* = 0.31 [0.12; 0.46]). Thus, the thought experiment does not change the main result that participants with depression increase their ratings with consummation. If anything, our repeated measures within constructs underestimate the effect compared to this scenario.

##### Anhedonia

To measure symptoms of anhedonia, we used the German version of the Snaith-Hamilton Pleasure Scale (SHAPS^160^^,^^161^), which is widely recognised as a measure of hedonic capacity and has been validated to measure anhedonia in clinical and research settings^162^ and is relevant for both, the scientific literature (where the SHAPS is commonly used), and clinical practice according to recent recommendations by researchers in the field.^163^ Participants indicated on 14 items how much they agreed or disagreed (Likert scale with 4 categories) with statements about experiencing pleasure over the last few days. The statements cover interests (e.g., “I would find pleasure in my hobbies and pastime”), social interactions (e.g., “I would enjoy seeing other people’s smiling faces”), sensory experiences (e.g., “I would enjoy a warm bath or refreshing shower”), and food (e.g., “I would be able to enjoy my favorite meal”). We calculated an overall sum score ranging from 0 (minimum, no anhedonia) to 42 points (maximum, anhedonia).

##### Depression severity

To measure depression severity, we used *Beck’s Depression Inventory II* (BDI-II), a well-validated and widely used self-report questionnaire to assess the severity of affective and somatic symptoms of depression in clinical and research settings (21 items;^164^^,^^165^). The sum of four items (loss of pleasure, loss of interest, loss of energy, and loss of sexual interest in sex) has been used as a sensitivity measure to describe anhedonia.^166^

##### Atypical depression

To measure the extent of atypical depression, we calculated the atypical balance score from the SIGH-ADS.^167^ The atypical balance score weights the atypical items (weight gain, appetite increase, increased eating, carbohydrate craving or eating, hypersomnia, fatigability, mood or energy dips, and social withdrawal) against overall symptom presence as follows: Total 8-items Atypical Symptoms score divided by the total 25-item SIGH-ADS score (i.e., 17-item Hamilton score + 8-item Atypical Symptom score), multiplied by 100. Thus, the atypical balance score represents the percentage of atypicality, ranging from 0 (minimum) to 100% (maximum). In our MDD sample, the scores were approximately normal distributed and ranged from 20 to 60 (mean = 40,21, median = 39,64, sd = 8,28). We stratified the MDD sample into participants with low atypical MDD (below median) versus high atypical MDD (above median), allowing to include the categorial Atypical Group Factor (HCP vs. melancholic MDD vs. atypical MDD) to test across the whole sample. We complemented the analysis using the atypical balance score as a continuous measure by setting the scores of HCPs to zero and group-centering the scores before including the atypical balance score as a continuous factor.

### Quantification and statistical analysis

#### Preprocessing

As a measure of insulin resistance, we calculated the homeostasis model assessment of insulin resistance (HOMA-IR) using fasting glucose and insulin levels (^168^, insulin [pmol/l]/6,945) ∗ glucose [mg/dl]/405) as well as the triglyceride–glucose (TyG) index using fasting triglyceride and glucose levels (^169^; Ln(triglycerides [mg/dl] ∗ glucose [mg/dl])/2). Since the distribution of hormone levels was skewed, data were log-transformed for parametric analyses, a common tool for biological data.^43^^,^^90^ Likewise, we log-transformed the HOMA-IR,^170^ whereas the TyG is already log-transformed per definition.^169^ Shapiro-Wilk tests for normality indicated approximately normal distributions for residualised log-transformed values of acyl ghrelin (*W* = 0.99, *p* = 0.77), des-acyl ghrelin (*W* = 0.98, *p* = 0.09), HOMA-IR (*W* = 0.99, *p* = 0.31), insulin (*W* = 0.99, *p* = 0.47), and glucose (*W* = 0.98, *p* = 0.29), but not for TyG (*W* = 0.96, *p* = 0.001). However, visual inspection and QQ-Plots indicate that TyG is approximately normally distributed. Additionally, the Kolmogorov-Smirnov tests indicate normal distribution as well (D = 0.07, *p* = 0.61).

To control for potential confounding effects of sex, age, and BMI, hormone values were residualised for these variables before being entered into the models.^171^^,^^172^ By using residuals, variance attributable to the linear effects of sex, age, and BMI is accounted for, ensuring that the observed hormone effects are incremental to such participant characteristics. Additionally, sex, age, and BMI were included as nuisance regressors in all models to control for their potential effect on the outcomes. While our study was not powered to test sex-specific interactions with, we report significant results for these nuisance regressors to inform future work.

#### Statistics for primary hypotheses

##### Models for repeated food reward ratings

For our first hypothesis of reduced wanting and/or liking ratings in MDD, we used two non-independent linear mixed-effects models using restricted maximum likelihood estimation (Model 1–2). Specifically, we modeled the dependent variables (wanting and liking) as outcome, using the following independent variables: Group (dummy coded, 0: HCP, 1: MDD), Phase (dummy coded, 0: anticipation, 1: consummation), and their interaction Group x Phase to test for group differences in relative changes between reward phases. Furthermore, we included Snack type (sum coded), and all models included BMI, age, and sex (centered) as potential confounders. To account for inter-individual variance in repeated ratings, we included random intercepts and slopes for snack type and phase. For our second hypothesis of altered liking but not wanting ratings in anhedonia, we replaced Group with SHAPS (centered, Model 3–4). To decide how to model the factor phase that captures ratings from anticipation to consummation we used model comparisons (S1). To verify the theory-driven distinction when anticipation changes into consummation, we used a data-driven approach, comparing three models with different categorizations of anticipation and consummation: (a) a 2-level dummy factor with phase 1 + 2 (food cues, and proximal sight/smell) vs. phase 3–5 (tasting); (b) a 3-level dummy factor with phase 1 (food cues), phase 2 (proximal sight/smell, and phase 3–5 (tasting); and (c) a 2-level dummy factor with phase 1 (food cues) and phase 2–5 (proximal sight/smell) and tasting). Model (c) outperformed the initial model (S1). Crucially, the winning model (anticipation vs. consummation, where consummation includes proximal sight, touching, and smelling of food) corresponds to the definition by Salamone and Correa (2012), which differentiates consummatory processes (‘to complete’) from consumption, reflecting the direct interaction with the goal of an instrumental response. Thus, we refer to the terminal stage of motivated behavior as the consummatory phase and refer to phase 1 as anticipation and phase 2–5 as consummation. Nevertheless, results do not differ qualitatively depending on the phase coding (Table S4).

##### Multivariate tests for associations of metabolic hormones and reward ratings

For our third hypothesis, we used multivariate regression to evaluate potential associations of liking and wanting ratings with ghrelin and insulin sensitivity while accounting for Group and Phase and the interaction of Group and phase. We also included age, sex, and BMI as covariates.

##### Bayesian analyses

According to Hypothesis 2, anhedonia is often conceptualized as a consummatory deficit. Given the prevalence of anhedonia being characterized as a “pleasure deficit” in the literature,^32^^,^^33^^,^^34^^,^^35^ especially in pre-clinical models,^19^^,^^20^ we aimed to highlight the strength of evidence our data provides against this hypothesis. Using Frequentist statistics, we can state whether data is unlikely to be observed given the null hypothesis. Based on *p*-values that quantify this, we can then reject or accept an alternative hypothesis (i.e., Neyman-Pearson approach). However, we cannot quantify the support for the null hypothesis or quantify the strength of support for the alternative hypothesis.^173^ In contrast, Bayesian methods provide a principled framework for evaluating both the evidence for an effect or its absence.^109^^,^^110^^,^^174^^,^^175^^,^^176^ This helps avoid the binary “significant/non-significant” interpretation and allows for more nuanced conclusions. In line with a recent review,^177^ we supplement our analyses with Bayesian tests to derive an estimate of the strength of evidence for or against specific hypotheses given the observed data. The Bayesian approach can directly evaluate the relative strength of evidence for null and alternative hypotheses by providing Bayes Factors (BF). A BF_+0_ of 3 indicates that the data is 3x more likely to happen under the alternative hypothesis than the null hypothesis. To evaluate the strength of evidence in rating changes across phases between groups (MDD vs. HCPs), we used Bayesian independent samples t-tests as implemented in JASP (JASP Team, 2024, v 0.18.3) using default effect size priors (Cauchy scale 0.707) (Robustness check S5). Changes in ratings and their correlation with SHAPS were analyzed using Bayesian correlation tests using a stretched beta prior with a width of 0.3 (Robustness check S5), as large correlations have rarely been found in psychological research (88,89). Likewise, to evaluate the strength of evidence of an anticipatory deficit, we repeated these tests for anticipatory ratings for MDD vs. HCPs and their association with the SHAPS.

#### Statistics for exploratory analyses

To explore additional factors that may inform future research, we conducted exploratory analyses. Specifically, we examined associations between metabolic hormone levels and MDD as well as anhedonia using linear models. We also tested whether depression subtypes influenced the primary associations. Finally, given that ghrelin was associated with both liking and wanting, we assessed whether it also moderated the coupling between liking and wanting.

#### Statistics for sensitivity analyses

In line with the concept of multiverse analyses,^178^ we test the robustness of the primary associations across multiple reasonable analytical choices by conducting several sensitivity analyses. These included: controlling for additional covariates such as depression severity, medication type, and substance use (Table S6); including liking ratings in wanting models (Table S2); applying an alternative operationalization of anhedonia (BDI-anhedonia); examining potential group differences in taste ratings; and reanalyzing the data using non-parametric methods (Table S3). Since we deliberately assessed both wanting and liking across all phases of the task—without enforcing that each construct maps exclusively onto one phase—we also tested whether results hold when assuming wanting is predominant during anticipation and liking during consummation.

#### Software and thresholds

Primary analyses were conducted with R (v4.3.2; R Core Team 2021). For statistical modeling, we used the *lmer* and *summary* function of the ‘*lmerTest’* package v3.1.3^179^ which estimates degrees of freedom using the Satterthwaite approximation. We report effect sizes using unstandardized beta coefficients with 95% confidence intervals.^180^ In addition, we report the commonly used effect size *r* with 95% confidence interval for primary hypotheses using the R package *effectsize* (function *t_to_r*) in the supplement (Table S5). For multivariate regression analysis, we used the *lm* and *anova* function. For wild bootstrapping, we used the *lmeresampler* package. For Bayesian tests, we used JASP (JASP Team, 2024, v 0.18.3). For our primary hypotheses, we considered α < 0.05 as significant. Exploratory analyses (a. depression subtype, b. group differences in hormones, c. SHAPS associations with hormones) were corrected by controlling the false discovery rate.^181^

#### Additional resources

Supplemental figures and tables are available as supplemental information. Additional full model outputs of main hypotheses tested can be found in the GitHub repository: https://doi.org/10.5281/zenodo.19499946.

This study is part of a larger clinical trial on the gut–brain axis in depression^95^^,^^182^ and is registered at ClinicalTrials.gov (Identifier: NCT05318924; https://clinicaltrials.gov/study/NCT05318924).

## Supplemental information

Document S1. Figures S1–S6 and Tables S1–S6

Document S2. Article plus supplemental information
