# Supplementary material for: Blunted anticipation, but not consummation, of food rewards in depression
Source: Cell Rep Med. 2026 May 12;7(6):102796. doi: 10.1016/j.xcrm.2026.102796 (PMC13293956; doi:10.1016/j.xcrm.2026.102796)
Supplement: Document S1. Figures S1–S6 and Tables S1–S6 [file mmc1.pdf]

Cell Reports Medicine, Volume *7*

Supplemental information

Blunted anticipation, but not consummation, of food rewards in depression

Corinna Schulz, Johannes Klaus, Franziska Peglow, Sabine Ellinger, Anne Kühnel, Martin Walter, and Nils B. Kroemer

Supplemental Figures

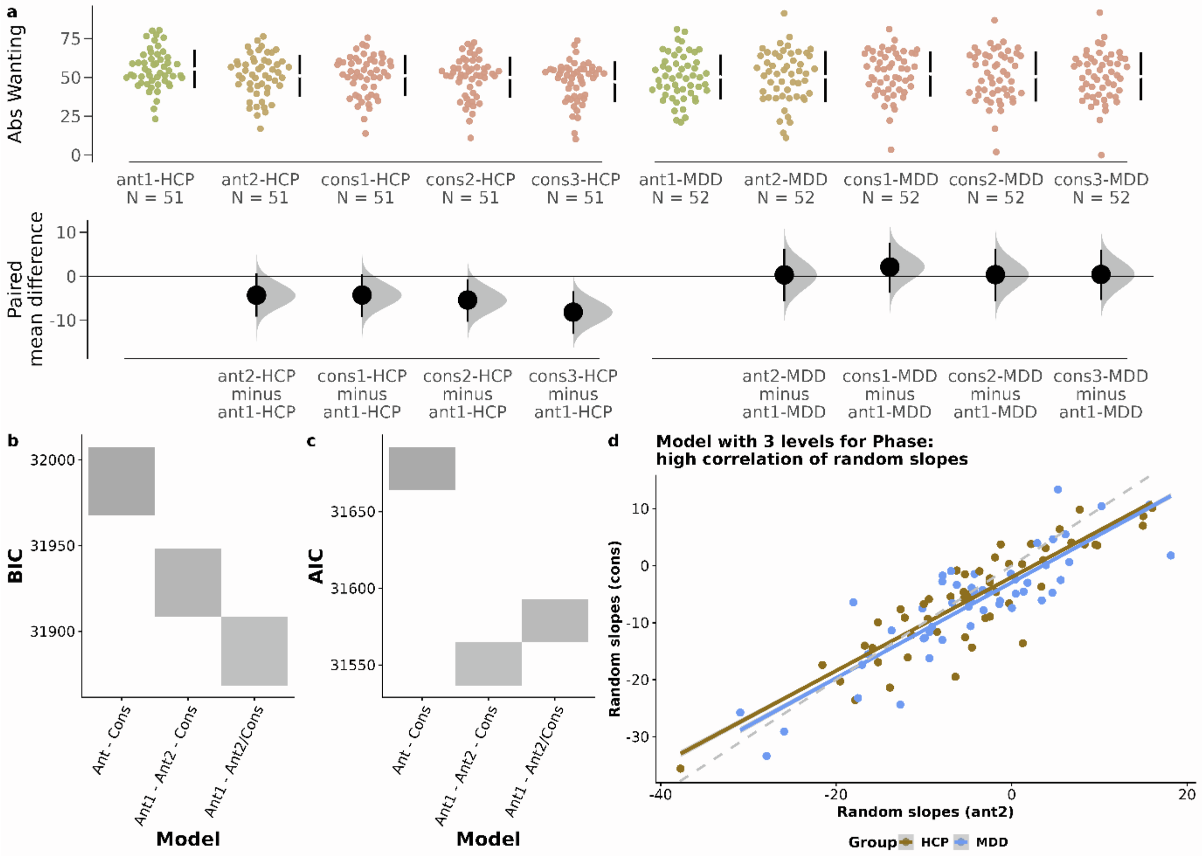

Fig. S1. Model comparison for coding of anticipatory and consummatory phases. **Related to Fig. 1B.** A. The taste test comprises 5 rounds moving gradually from anticipation to consummation. B. We compared 3 variants to model the factor "phase". (1) A 2-level factor with the first two phases (food cues, sight and smell of snacks) as one anticipatory phase, and the last three phases (repeated consummation) as one consummatory phase [Ant-Cons]. (2) A 3-level factor (1st anticipation, 2nd anticipation, consummation) [Ant1-Ant2-Cons] and (3) as a 2- level phase factor (1st anticipation, 2nd anticipation/consummation) [Ant1-Ant2/Cons]. The latter two models performed better than the first in a model comparison using the Bayesian and Akaike Information criteria, indicating a better fit-complexity trade-off. C. We found that the random slopes correlation for the 3-level phase factor was very high (r = 0.83), suggesting that the additional third phase does not differ qualitatively. Therefore, we used the 2-level phase factor to separate first anticipation (i.e., cued) from later anticipation (i.e., sight and smell) and consummation for all further analysis. Importantly, the conclusions for the group differences did not change qualitatively using different phase coding

.

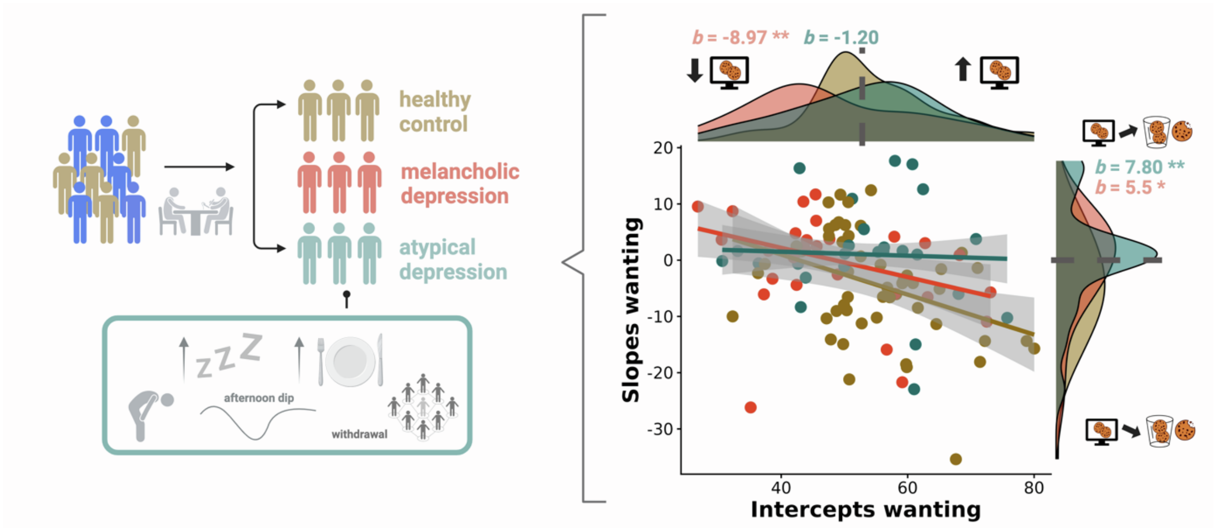

Fig. S2. Lower wanting in melancholic MDD. **Related to Fig. 1**. MDD is a heterogeneous condition, and patients may experience increases or decreases in appetite and body weight during a depressive episode, with melancholic depression being characterized by decreased appetite. To assess the potential influence of depression subtype and to inform future studies, we stratified the MDD sample into participants with low atypical MDD (below median atypical balance score) versus high atypical MDD (above median atypical balance score), allowing to include the categorial *Atypical Group Fact*or (HCP vs. low atypical balance vs. high atypical balance) to test across the whole sample and report here, whether results change using the depression subtype. We complemented the analysis using the atypical balance score as a continuous measure by setting the scores of HCPs to zero and group-centering the scores before including the atypical balance score as a continuous factor. Patients with more melancholic MDD primarily reported blunted food wanting during anticipation (vs. HCP: *b* = -8.97, [CI: -14.95; -2.99], p_{FDR} = .012, vs. more atypical MDD: *b* = 7.77, [CI: 0.80; 14.73], p_{FDR} = .07, Fig. S3). In contrast, patients with more atypical MDD did not initially show reduced food wanting (vs HCP: *b* = -1.20, [CI: -7.30; 4.91], p_{FDR} = .70). Despite differences in initial ratings, all patients with MDD showed comparable increases in wanting during the consummatory phase (atypical: $b_{GroupxPhase}$ = 7.80, [CI: 2.15; 13.44], p_{FDR} = .012 vs. more melancholic MDD: $b_{GroupxPhase}$ = 5.73, [CI: 0.23; 11.24], p_{FDR} = .044). Consequently, patients with more atypical MDD even reported higher wanting during consummation compared to HCPs, however, this did not survive control for false discovery rate (*b* = 6.60, [CI: 0.83; 12.37], p_{FDR} = .081). In contrast, we did not find differences in liking between more melancholic (*b* = -2.05, [CI: -10.22; 6.11], p_{FDR} = .93) and more atypical MDD (*b* = 2.54, [CI: -5.80; 10.88], p_{FDR} = .93) compared to HCPs, or between depression subtypes (*b* = -4.60, [CI: -14.10; 4.91], p_{FDR} = .62). For participants with MDD extend of atypical symptoms were evaluated using the atypical balance score from the SIGH-ADS. Lower wanting during cued anticipation was driven by participants with melancholic (b = -8.97, p = .004) and not atypical MDD (b = -1.20, p = .70). Both, melancholic (b = 5.73, p = .044) and atypical MDD (b = 7.80, p = .008) increased their wanting ratings after cued anticipation.

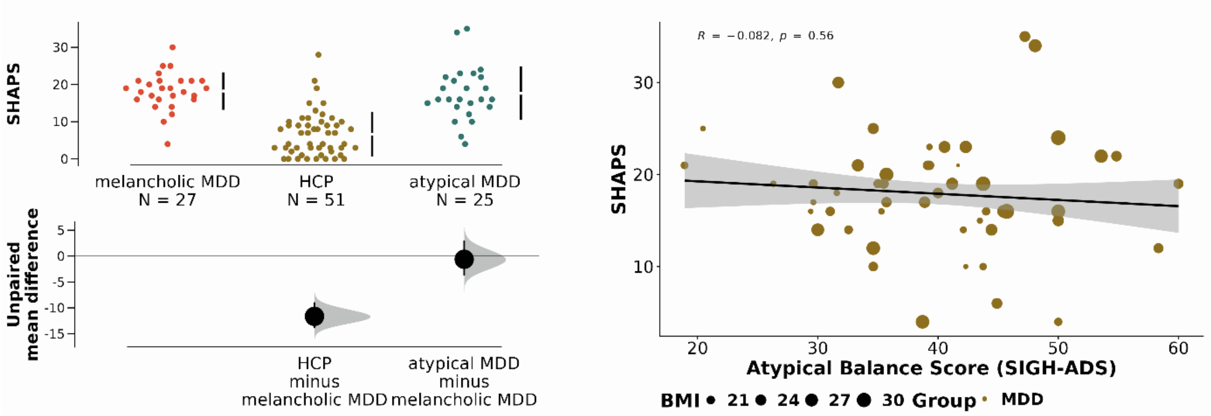

Fig. S3. SHAPS does not differ between depression subtypes. **Related to Fig. 2 and S2.** Cumming estimation plots show no difference in SHAPS ratings between melancholic and atypical MDD (left). Effect size and bootstrapped 95% CIs are plotted below the raw data. Within participants with depression, atypical balance score was also not associated with SHAPS (r = -.082, p = .56, right).

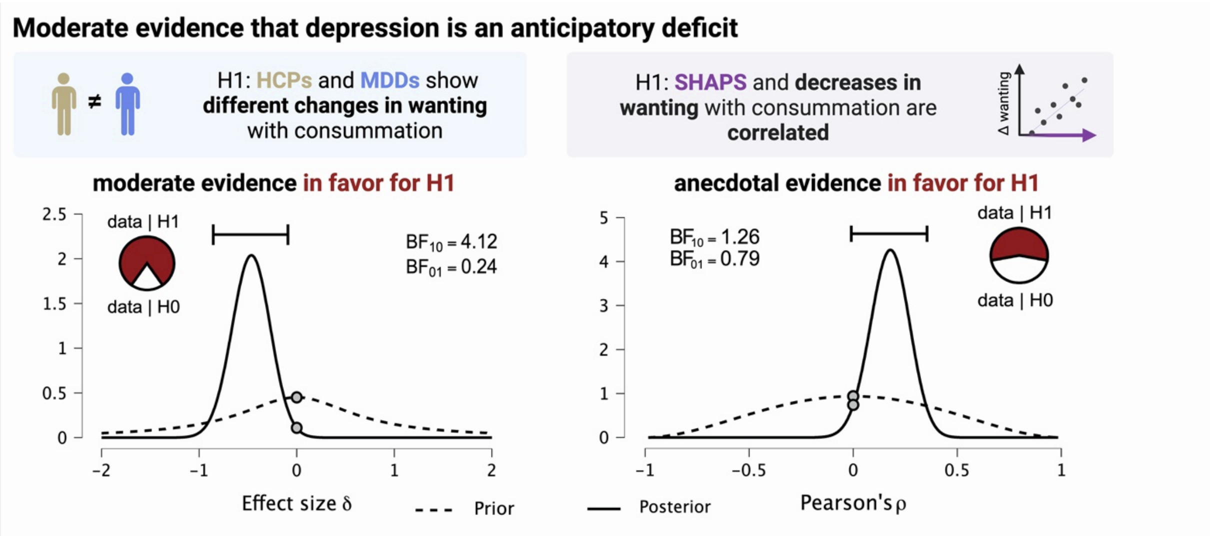

Fig. S4. Depression as anticipatory but not consummatory deficit. Related to Fig. 3. Bayesian hypothesis testing showing moderate evidence for the hypothesis that participants with MDD (vs HCPs) show different wanting changes from anticipation to consummation (left panel; two-sided Bayesian independent samples *t*-test). Anecdotal evidence for the hypothesis that SHAPS (i.e., lower "hedonic tone") is associated with stronger wanting decreases during consummation (bottom panel; Bayesian Correlation). *BF* = Bayes factor (with levels of evidence: 1-3 anecdotal, 3-10 moderate, 10-30 strong). A probability wheel on an area of size 1 represents the BF10, respectively. Created with BioRender.com

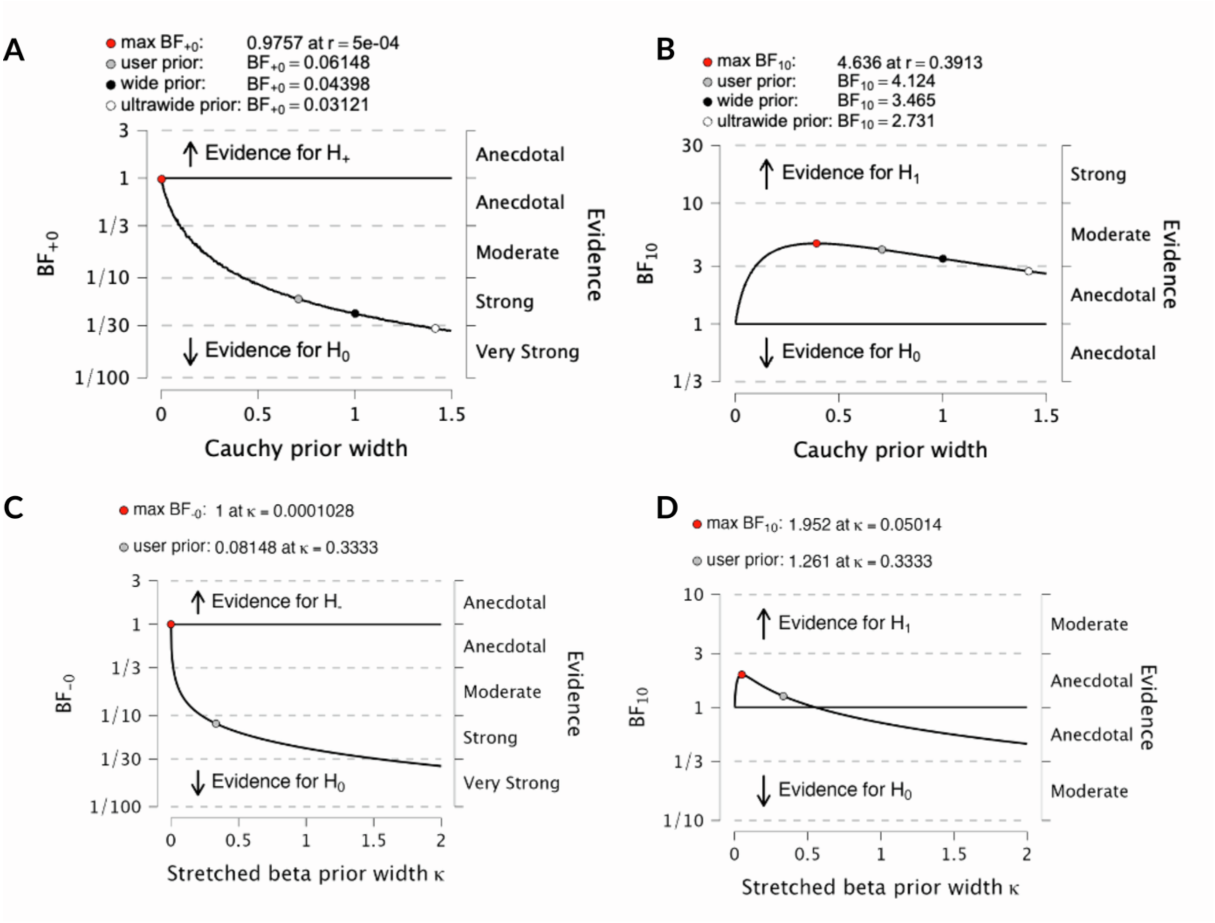

Fig. S5. Robustness Check for the Bayesian Tests. Related to Fig. 3. And S4. A. Robustness check for the one-sided Indedendent Samples T-Test that HCPs show greater wanting during consummation than patients with MDD compared to anticipation. B. Robustness check for the two-sided Independent Samples T-Test that HCPs and patients with MDD differ in their wanting adjustments during consummation. C. Robustness Check for the Bayesian directed Correlation that SHAPS is negatively associated with changes in wanting with consummation. D: Robustness check for the correlation test whether SHAPS is correlated with changes in wanting with consummation.

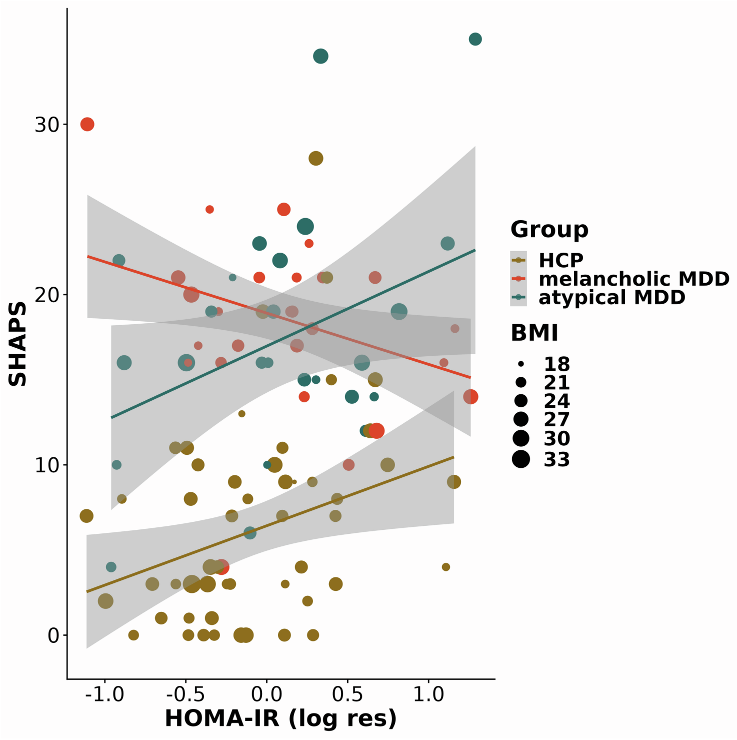

Fig. S6. HOMA-IR and SHAPS depending on depression subtype. Related to Fig. 2. A. Robustness check for the one-sided Indedendent Samples T-Test that HCPs show greater wanting during consummation than patients with MDD compared to anticipation. B. Robustness check for the two-sided Independent Samples T-Test that HCPs and patients with MDD differ in their wanting adjustments during consummation. C. Robustness Check for the Bayesian directed Correlation that SHAPS is negatively associated with changes in wanting with consummation. D: Robustness check for the correlation test whether SHAPS is correlated with changes in wanting with consummation.

Supplemental Tables

Table S1. Participant comorbidities. Related to Table 1.

Characteristic	HCP (N=51)	MDD (N=52)	Overall (N=103)
**Obsessive compulsive disorder (lifetime)**	1 (2.0%)	5 (9.6%)	6 (5.8%)
**Post-traumatic stress disorder (lifetime)**	1 (2.0%)	4 (7.7%)	5 (4.9%)
**Attention Deficit Disorder**			
Inattentive type	4 (7.8%)	1 (1.9%)	5 (4.9%)
Combined type	0 (0%)	4 (7.7%)	4 (3.9%)
**Binge eating disorder (lifetime)**	1 (2.0%)	1 (1.9%)	2 (1.9%)
**Alcohol substance use disorder**			
Lifetime	7 (13.7%)	8 (15.4%)	15 (14.6%)
Severity: moderate (else light)	0 (0%)	2 (3.8%)	2 (1.9%)
Current	3 (5.9%)	6 (11.5%)	9 (8.7%)
**Other substance use disorder**			
Lifetime	2 (3.9%)	4 (7.7%)	6 (5.8%)
Severity: moderate (else light)	1 (2.0%)	1 (1.9%)	2 (1.9%)
Current	2 (3.9%)	2 (3.8%)	4 (3.9%)
**Social anxiety**		11 (21.2%)	11 (10.7%)
**Generalized anxiety**		6 (11.5%)	6 (5.8%)
**Panic disorder**		8 (15.4%)	8 (7.8%)

Note. Listed are comorbidities that were no exclusion criteria. Current refers to fulfilment of diagnostic criteria within the last 12 months. Severe substance abuse was excluded. Social anxiety, generalized anxiety, and panic disorder are only mentioned for MDD to strengthen that they were exclusion criteria for HCPs. Values are counts with percentage of respective group.

Table S2. Including Liking in the Wanting Models does not render main conclusions

model	term	estimate	Std. Error	p-value
**MDD and Wanting**	MDD	**-5.01**	**2.32**	**.03**
	Liking	**0.19**	**0.05**	**.0005**
	MDD * Liking	-0.01	0.07	.85
	MDD * Phase	**6.12**	**2.16**	**.005**
	Liking * Phase	**0.18**	**0.04**	**<.001**
	Liking * Phase * MDD	-0.11	0.06	.067
**Anhedonia and Wanting**	SHAPS	**-0.38**	**0.14**	**.008**
	Liking	**0.18**	**0.03**	**<.001**
	SHAPS * Liking	-0.006	0.004	.16
	SHAPS * Phase	**0.29**	**0.13**	**.035**
	Liking * Phase	**0.12**	**0.03**	**<.001**
	Liking * Phase * SHAPS	0.004	0.004	.33

Table S3. Bias of non-parametric method using wild bootstrapped results for linear mixed effects models (N_B = 1000).

model	term	test	estimate	se	lower	upper
**Liking and depression**	MDD	P	0.21	3.44	-6.54	6.96
		NP	0.21	3.29	-6.32	6.56
		**bias**	**0**	**+0.15**		
	MDD* Phase[consummation]	P	2.65	2.33	-1.91	7.23
		NP	2.66	2.32	-1.75	7.34
		**bias**	**+0.01**	**-0.01**		
**Liking and SHAPS**	SHAPS	P	-0.36	0.20	-0.76	0.04
		NP	-0.36	0.21	-0.78	0.06
		**bias**	**0**	**+0.01**		
	SHAPS* Phase[consummation]	P	0.25	0.14	-0.03	0.52
		NP	0.25	0.13	-0.004	0.52
		**bias**	**0**	**-0.01**		
**Wanting and depression**	MDD	P	-5.17	2.56	-10.19	-0.15
		NP	-5.17	2.64	-10.40	-0.08
		**bias**	**0**	**-0.08**		
	MDD* Phase[consummation]	P	6.73	2.32	2.19	11.28
		NP	6.74	2.41	2.17	11.60
		**bias**	**+0.01**	**-0.09**		
**Wanting and SHAPS**	SHAPS	P	-0.40	0.15	-0.70	-0.10
		NP	-0.40	0.17	-0.73	-0.08
		**bias**	**0**	**+0.02**		
	SHAPS* Phase[consummation]	P	0.30	0.14	0.02	0.58
		NP	0.30	0.13	0.06	0.57
		**bias**	**0**	**-0.01**		

Note. Wild bootstrapping makes no distribution assumptions and allows for heteroskedasticity[1]. For the four main models, we show the terms of interest (i.e., fixed effects for MDD or SHAPS and their interaction with phase), the coefficient (estimate), the standard error (se) and the 95% confidence interval (upper, lower) for the parametric test (P) and the non-parametric (NP) bootstrapping. The bias of the parametric method was determined by calculating the difference to the non-parametric parameter estimate and standard error.

Table S4. Model results for liking and wanting with depression using 3-factor coding

Model	Term	Estimate	Std. error	p-value
Wanting	MDD	**-5.22**	**2.56**	**.045**
	MDD*Phase[anticipation2]	4.66	2.63	.079
	MDD*Phase[consumption]	**7.43**	**2.36**	**.002**
	SHAPS	**-0.40**	**0.15**	**.0097**
	SHAPS*Phase[anticipation2]	0.22	0.16	.17
	SHAPS*Phase[consumption]	**0.25**	**0.009**	**.027**
Liking	MDD	0.21	3.44	.95
	MDD*Phase[anticipation2]	1.77	2.49	.48
	MDD*Phase[consumption]	2.96	2.40	.22
	SHAPS	-0.36	0.20	.081
	SHAPS*Phase[anticipation2]	0.15	0.15	.32
	SHAPS*Phase[consumption]	**0.28**	**0.14**	**.054**

Table S5. Similar results for wanting using the 3-level coding of the factor phase. Results are shown for using three phases (ant1 – ant2 – consummation). Results are highlighted in green when they correspond to the results reported using the winning model (Ant1 – Ant2/consummation; reported main manuscript). The table shows that the conclusions derived in the manuscript do not depend critically on choosing the Ant1 – Ant2/consummation model over the (ant 1 – ant2 – consummation).

Table S5. Standardized effect size *r* for main results

Model	Term	*b*	*p*-value	Correlation coefficient *r* and 95% CI
**Liking and depression**	MDD	0.21	.95	0.01 [-0.19; 0.20]
	MDD* Phase [consummation]	2.65	.26	0.11 [-0.08; 0.30]
**Liking and SHAPS**	SHAPS	-0.36	.081	-0.18 [-0.36; 0.02]
	SHAPS* Phase [consummation]	0.25	.080	0.17 [-0.02; 0.35]
**Wanting and depression**	MDD	-5.17	**.046**	-0.20 [-0.37; 0.003]
	MDD* Phase [consummation]	6.73	**.004**	0.28 [0.09; 0.44]
**Wanting and SHAPS**	SHAPS	-0.40	**.010**	-0.26 [-0.42; -0.06]
	SHAPS* Phase [consummation]	0.30	**.037**	0.21 [0.01; 0.38]

Table S6. Main results when excluding individuals with substance abuse

		Entire sample (N = 103)		Exclude any SUD in HCPs (N =94)		Exclude AUDIT >= 15 in HCP and MDD (N =100)	
**Model**	**Term**	***b***	***p*-value**	***b***	***p*-value**	***estimate***	***p*-value**
**Liking and MDD**	MDD	0.21	.95	0.31	.93	0.47	.89
	MDD* Phase [consummation]	2.65	.26	3.06	.21	2.15	.36
**Liking and SHAPS**	SHAPS	-0.36	.081	-0.41	.059	-0.34	.11
	SHAPS* Phase [consummation]	0.25	.080	0.29	**.046**	0.22	.12
**Wanting and MDD**	MDD	-5.17	**.046**	-4.91	.072	-5.29	**.049**
	MDD* Phase [consummation]	6.73	**.004**	6.07	**.016**	6.60	**.006**
**Wanting and SHAPS**	SHAPS	-0.40	**.010**	-0.42	**.010**	-0.40	**.012**
	SHAPS* Phase [consummation]	0.30	**.037**	0.30	**.048**	0.28	.055

Note. To test whether main results reported in the manuscript are influenced by substance use we provide this table. We first excluded all HCPs meeting DSM-5 criteria for any current SUD, resulting in a reduced control group (n = 94). This improved the clinical definition of the HCP group but introduced greater imbalances between groups, as MDD participants with mild or moderate SUDs were retained to reflect real-world comorbidity.To account for this asymmetry, we conducted a second analysis using the Alcohol Use Disorders Identification Test (AUDIT) scores. Participants scoring ≥15 (suggesting probable alcohol dependence) were excluded regardless of group, resulting in a sample of 100 participants. Importantly, both sensitivity analyses yielded results consistent with the original models, especially for our primary outcome: wanting ratings. The Group × Phase interaction remained significant across all models. Minor fluctuations in p-values for some terms are noted in the Table below, but none of these alter the interpretation of the findings. These analyses support the robustness of our main conclusions and confirm that the inclusion of participants with mild or moderate SUDs did not drive the observed group effects. Differences in significance to the original analysis are highlighted in orange.

References

1. Modugno, L., and Giannerini, S. (2015). The Wild Bootstrap for Multilevel Models. Commun. Stat. - Theory Methods *44*, 4812–4825. https://doi.org/10.1080/03610926.2013.802807.
